# Identification of Bovine miRNAs with the Potential to Affect Human Gene Expression

**DOI:** 10.3389/fgene.2021.705350

**Published:** 2022-01-11

**Authors:** Moldir Myrzabekova, Siegfried Labeit, Raigul Niyazova, Aigul Akimniyazova, Anatoliy Ivashchenko

**Affiliations:** ^1^ Faculty of Biology and Biotechnology, Al-Farabi Kazakh National University, Almaty, Kazakhstan; ^2^ Medical Faculty Mannheim, University of Heidelberg, Heidelberg, Germany; ^3^ Myomedix GmbH, Neckargemuend, Germany

**Keywords:** miRNA, exogenous miRNA, mRNA, gene, Bos taurus, human, disease

## Abstract

Milk and other products from large mammals have emerged during human evolution as an important source of nutrition. Recently, it has been recognized that exogenous miRNAs (mRNA inhibited RNA) contained in milk and other tissues of the mammalian body can enter the human body, which in turn have the ability to potentially regulate human metabolism by affecting gene expression. We studied for exogenous miRNAs from *Bos taurus* that are potentially contain miRNAs from milk and that could act postprandially as regulators of human gene expression. The interaction of 17,508 human genes with 1025 bta-miRNAs, including 245 raw milk miRNAs was studied. The milk bta-miR-151-5p, bta-miR-151-3p, bta-miRNA-320 each have 11 BSs (binding sites), and bta-miRNA-345-5p, bta-miRNA-614, bta-miRNA-1296b and bta-miRNA-149 has 12, 14, 15 and 26 BSs, respectively. The bta-miR-574-5p from cow’s milk had 209 human genes in mRNAs from one to 25 repeating BSs. We found 15 bta-miRNAs that have 100% complementarity to the mRNA of 13 human target genes. Another 12 miRNAs have BSs in the mRNA of 19 human genes with 98% complementarity. The bta-miR-11975, bta-miR-11976, and bta-miR-2885 BSs are located with the overlap of nucleotide sequences in the mRNA of human genes. Nucleotide sequences of BSs of these miRNAs in 5′UTR mRNA of human genes consisted of GCC repeats with a total length of 18 nucleotides (nt) in 18 genes, 21 nt in 11 genes, 24 nt in 14 genes, and 27–48 nt in nine genes. Nucleotide sequences of BSs of bta-miR-11975, bta-miR-11976, and bta-miR-2885 in CDS mRNA of human genes consisted of GCC repeats with a total length of 18 nt in 33 genes, 21 nt in 13 genes, 24 nt in nine genes, and 27–36 nt in 11 genes. These BSs encoded polyA or polyP peptides. In only one case, the polyR (*SLC24A3* gene) was encoded. The possibility of regulating the expression of human genes by exogenous bovine miRNAs is discussed.

## Introduction

The miRNAs (mRNA inhibited RNA) are 18–24-nucleotide-long RNA nanoscale molecules that are highly conserved among species. They regulate post-transcriptional gene expression either by inhibiting mRNA translation or by degrading through exonuclease action (Huntzinger and Izaurralde, 2011; Fabian and Sonenberg, 2012; Ipsaro and Joshua-Tor, 2015; Jonas and Izaurrallde, 2015). In recent years, numerous studies have shown that the milk of humans and cows is enriched with miRNAs (*Chen et al., 2010*; *Weber et al., 2010*), most of which are packed in extracellular vesicles that are 30–120 nm in diameter, namely exosomes, which are derived from all types of cells and released into all biological fluids, such as blood plasma, serum, urine, breast milk, colostrum, and more (*Kosaka et al., 2010*; *Gu et al., 2012*; *Xiao et al., 2018*; *Yun et al., 2021*; *Zeng et al., 2020*; *Zeng et al.,2021*). Zhang et al. suggested that exogenous miRNAs (ex-miRs), specifically from plants, can withstand the digestion process, enter the animal’s bloodstream through the gastrointestinal tract, and regulate gene expression (*Zhang L. et al., 2012* *,* *Zhang et al., 2012 Y.*; *Dickinson et al., 2013*; *Laubier* et *al., 2015*; *Title et al., 2015*; *Auerbach et al., 2016*; *Rakhmetullina et al., 2020*). It was shown that when piglets were fed pig or cow’s milk, miRNAs could be absorbed both *in vivo* and *in vitro*, which creates the basis for understanding the participation of miRNAs in physiological functions *(* *Lin et al., 2020* *).* miRNAs are key effectors in physiology and development of infants (*Wang L. et al., 2021*; *Zhou Q. et al., 2021*; *Leroux et al., 2021*; *Miao et al., 2021*; *Shah et al. 2021* *).* In earlier works, it was shown that ex-miRs from food are bioactive, and both humans and animals can absorb miRNAs from a diet of plant or animal source (*Zhang L. et al., 2012*; *Wang et al., 2012*; *Baier et al., 2014*; *Zhou et al., 2015*). Baier et al. provided evidence that the amounts of miRNAs absorbed from milk are sufficient to alter human gene expression, i.e., miRNAs from one mammalian species can affect gene networks in another species (*Zhou et al., 2012*; *Baier et al., 2014*). Milk exosomes increase the stability of miRNAs, which facilitates their absorption through the digestive tract *(* *Aarts et al., 2021*; *Diomaiuto et al., 2021*; *Gao et al., 2021*; *Marsh et al., 2021*; *Wehbe & Kreydiyyeh, 2021* *)*. Animal and plant miRNAs are detected in all foods irrespective of processing (*Dever et al., 2015*; Benmoussa and Provost, 2019; *Mar-Aguilar et al., 2020*; *Melnik et al., 2021*). The study on the miRNA content of milk was undertaken by Izumi et al. (2012); they found that colostrum contained twice the amount of miRNAs found in mature milk, and that immune- and development-related miRNAs had significantly higher levels of expression (*Izumi et al., 2012* *; Link et al., 2019)*. The authors demonstrated that miRNAs and messenger RNAs that exist naturally in milk were resistant to acidic conditions and RNAses, as well as to industrial processing conditions. Humans absorb biologically meaningful amounts of miRNAs from nutritionally relevant doses of cow’s milk; these miRNAs enter peripheral blood mononuclear cells and presumably other peripheral tissues, and physiological concentrations of milk miRNAs may affect human gene expression *in vivo* and in cell cultures (*Baier et al., 2014*; Lukasik and Zielenkiewicz, 2014; *Shu et al.*, *2015*; *Yim et al., 2016*).

Research in the field of dietary miRNAs has shifted away from miRNAs in plant-borne foods (*Rakhmetullina et al., 2020*) to miRNAs in foods of animal origin, particularly cow’s milk, after observations that a large proportion of milk miRNAs is encapsulated in extracellular vesicles such as exosomes. The miRNAs encapsulated in milk exosomes are stable under harsh conditions, such as low pH and exposure to ribonucleases (*Kosaka et al., 2010*; *Izumi et al, 2012*).

Furthermore, it has been established that human exosomes can pass the gastrointestinal mucosa and deliver their miRNA to various peripheral tissues, possibly regulating their target genes (*Gu et al., 2012*; *Kusuma et al., 2016*; *Munagala et al., 2016*; *Bayraktar et al., 2017*; *Bahrami et al., 2018*; Rajagopal and Harikumar, 2018; *Xiao et al., 2018*; *Komine-Aizawa et al., 2020*).

Breast milk is the main source of nutrition and supply of the child, containing the biologically active substances that regulate the development of the body (*Kim et al., 2020*; McNeill and Hirschi, 2020)*.* A significant part of regulatory molecules, including miRNAs, is transported in exosomes that are resistant to the conditions of the gastrointestinal tract, enter the bloodstream and spread throughout the recipient’s body (*Reif et al., 2020*; *Akimniyazova et al., 2021*; *Melnik B., 2021*; *Billa et al., 2021*; *Melnik B. C., 2021*; *Wang X. et al., 2021*; *Jiang et al., 2021*; *Leroux et al., 2021*; *Lowry et al., 2021*; *Xia et al., 2021*).

Since miRNAs have long been considered as exclusively endogenously acting molecules, miRNA exocytosis, secretion, and possible cross-species signaling roles have not been a focus of research during the past decade (*Wang et al., 2012*; *Baier et al., 2014*; Lukasik and Zielenkiewicz, 2014; *Bryniarski et al., 2015*; *Munagala et al., 2016*; *Yim et al., 2016*; *Golan-Gerstl et al., 2017*; *Manca et al., 2018*; *van Herwijnen et al., 2018*; *Zempleni et al., 2018*). So far, only a few studies have explored whether humans can absorb a meaningful amount of certain exosomal miRNAs from cow’s milk. The findings indicated that milk-derived miRNAs in pasteurized milk are absorbed by adults in meaningful amounts, and moreover, that endogenous miRNA synthesis cannot compensate for dietary deficiency (*Baier et al., 2014*; *Zhang et al., 2021*).

The high degree of sequence conservation of miRNAs between different mammalian species also suggests conserved functional roles of the miRNA-mRNA signal networks of orthologous genes. This is evidence of the similarity of the systems regulating the expression of mammalian genomes. The conservation of exogenous miRNAs, firstly, indicates the similarity of the systems for regulating the expression of genes and mammalian genomes and, secondly, allows manipulating miRNA changes as biocompatible regulators of biological processes. The aim of this work was to predict possible bovine miRNAs - human gene expression regulatory networks *in silico*. The obtained results will help to use exogenous miRNAs to purposefully change the expression of human genes.

## Materials and Methods

The nucleotide sequences of the 17 thousand mRNAs of targeted genes were downloaded from NCBI GenBank (http://www.ncbi.nlm.nih.gov accessed on 5 January 2020). The nucleotide sequences of the miRNAs were taken from miRBase v.22 (http://www.mirbase.org/ accessed on 5 January 2020). 1025 miRNAs encoded by the bovine genome are available in the miRBase database (https://www.mirbase.org/summary.shtml?org=bta). The miRNA BSs (binding sites) in the mRNAs of several genes were predicted using the MirTarget program (*Ivashchenko et al., 2014*; *Ivashchenko et al., 2016*). This program defines the following features of miRNA binding to mRNA: *1*) the initiation of the miRNA binding to the mRNAs from the first nucleotide of the mRNAs; *2*) the localization of the miRNA BSs in the 5′-untranslated region (5′UTR), coding domain sequence (CDS), and 3′-untranslated region (3′UTR) of the mRNAs; *3*) the schemes of nucleotide interactions between miRNAs and mRNAs *4*) the free energy of the interaction between miRNA and the mRNA (ΔG, kJ/mole); and the ratio ΔG/ΔGm (%) is determined for each site (ΔGm equals the free energy of the miRNA binding with its fully complementary nucleotide sequence). The MirTarget program finds hydrogen bonds between adenine (A) and uracil (U), guanine (G) and cytosine (C), G and U, and A and C. The free energy of interactions (ΔG) a pair of G and C is equal to 6.37 kJ/mol, a pair of A and U is equal to 4.25 kJ/mol, G and U, A and C equal to 2.12 kJ/mol (Friedman and Honig, 1995). The distances between the bound A and C (1.04 nm) and G and U (1.02 nm) are similar to those between bound G and C, A and U, which are equal to 1.03 nm (*Kool, 2001*; *Leontis et al., 2002*; *Garg and Heinemann, 2018*). The numbers of hydrogen bonds in the G–C, A–U, G–U, and A–C interactions were 3, 2, 1, and 1, respectively. By comparison, MirTarget differs from other programs in terms of finding the BSs of miRNA on the mRNAs of plant genes (*Dai et al., 2011*) in that *1*) it takes into account the interaction of the miRNA with mRNA over the entire miRNA sequence; *2*) it takes into account non-canonical pairs G–U and A–C; and *3*) it calculates the free energy of the interaction of the miRNA with mRNA, and when two or more miRNAs are bound with one mRNA or, if the BSs of two different miRNAs coincide in part, the preferred miRNA binding site is considered to be the one for which the free binding energy is greater. The adequacy of the program in terms of finding BSs has been confirmed in several publications (*Davis et al., 2005*; *Wang J. et al., 2016*, *Wang et al., 2016 Y.*; *Yurikova et al., 2019*). The MirTarget program predicts the BSs of plant and animal miRNAs equally well (*Bari et al., 2013* *,* *2014*). To construct WebLogo schemes, Create Sequence Logos were used (https://weblogo.berkeley.edu/logo.cgi). A better confirmation of the obtained results than “wet” experiments is provided by the schemes of interaction of nucleotides along the entire length of the miRNAs and BSs. There are no “wet” experiments to find BSs for all miRNAs nucleotides with BSs and to determine the free energy of their interaction. The existing programs for determining BSs based only on “seed,” in principle, cannot give adequate predictions of target genes and, moreover, the free energy of interaction of all nucleotides of miRNAs and BSs. In addition, widely used programs do not take into account the interaction of non-canonical nucleotide pairs, which significantly distorts the value of the free energy of interaction between miRNAs and BSs. Consideration of any of the above schemes shows which nucleotides of non-canonical pairs and in which position decrease the maximum possible energy of interaction between miRNAs and BSs. The schemes can be verified manually by finding the predicted miRNA BSs in the mRNA nucleotide sequence in the NCBI.

## Results

For mammalian predators, exogenous miRNAs can be ingested with raw food, and this pathway of miRNA transmission is preserved in nature during evolution. Some human food is prepared from meat without heat treatment, which does not lead to the destruction of miRNA.

### The Interaction of Milk Bta-miRNAs with mRNAs of Human Genes

The results of the possible influence of miRNAs from milk presented in Supplementary Table S1 shows, that out of 245 milk bta-miRNAs (*Chen et al., 2010*), 103 miRNAs can affect human protein synthesis. Consequently, the obtained data made it possible to select for future studies those miRNAs for which their target effect on human genes is known. Note that some miRNAs have more than 10 target genes. Interestingly, bta-miR-151-5p and bta-miR-151-3p, originating from the same pre-miRNA, each have 11 target genes, which may indicate the optimization of the energy costs of the synthesis of these miRNAs and the dependence of their target genes on a single source. The bta-miRNA-320, bta-miRNA-345-5p, bta-miRNA-614, bta-miRNA-1296b and bta-miRNA-149 have 11, 12, 14, 15 and 26 target genes, respectively. Consequently, these miRNAs have an increased effect on the expression of the human genome. The large numbers of human genes that can be targets of dairy bta-miRNAs indicate the possibility of using cow’s milk for baby food.

We identified bta-miR-574-5p from cow’s milk which had from one to 14 repeating BSs in mRNAs of 209 human genes (Supplementary Table S2). The bta-miR-574-5p and human miR-574-5p have identical nucleotide sequences, which, firstly, indicates that this miRNA is necessary for fetal development in the prenatal and postnatal periods, and, secondly, it controls the expression of a significant number of genes in the genome of cow and human. Since miR-574-5p is expressed in 17 genomes of other mammals (miRBase), its biological role is great in these organisms. The high value of ∆G from −115 kJ/mol to −123 kJ/mol indicates a significant interaction between miRNAs and BSs. The value of ∆G/∆Gm for associations of bta-miR-574-5p and *GABRB2, LRTM2, UBN2, SLITRK3* genes reaches 97%. The mRNA of the *SLITRK3* gene contains 12 BSs, which indicates a high probability of its interaction with bta-miR-574-5p.

We have selected 28 human target genes for bta-miR-574-5p, having the clusters of 14 or more BSs (Table 1). The bta-miR-574-5p and mRNA associations of these target genes had similar characteristics of the free energy of interaction. The increased number of BSs allows all of these mRNAs to interact with two bta-miR-574-5p at once, since the length of a cluster of 14 repeats is 50 nt with a bta-miR-574-5p length of 24 nt. These 28 bovine genes, like human target genes, also have BSs for miR-574-5p (Table 1). The obtained results indicate a strong dependence of the expression of many human genes on bta-miR-574-5p. One of the putative functions of miRNAs acting on many genes is to maintain their consistent expression. For example, increased expression of a gene will cause miR-574-5p to bind and decrease its concentration, which will lead to increased expression of other genes from the miR-574-5p target genes sample.

**TABLE 1:** Characteristics of interactions of bta-miR-574-5p with human mRNA 28 genes containing clusters of 14 and more repetitive BSs.

ID of human genes	Gene	ID of bovine genes	Start of first and last sites, nt	ΔG, kJ/mole	∆G/∆Gm, %
ID:90416	*CCDC32*	ID: 506935	861–903 (22)	−115÷−119	90–93
ID: 959	*CD40LG*	ID: 282387	1550–1578 (15)	−119	93
ID: 2033	*EP300*	ID: 112446776	8556–8587 (16)	−115÷−119	90–93
ID: 1112	*FOXN3*	ID: 505469	2419–2445 (14)	−115÷−119	90–93
ID: 2674	*GFRA1*	ID: 534801	8452–8480 (15)	−119	93
ID: 2736	*GLI2*	ID: 510255	6118–6147 (15)	−115÷−119	90–93
ID: 2742	*GLRA2*	ID: 537660	2525–2567 (20)	−115÷−119	90–93
ID: 22801	*ITGA11*	ID: 523755	4599–4635 (22)	−115÷−119	90–93
ID: 84056	*KATNAL1*	ID: 537739	4197–4238 (20)	−115÷−119	90–93
ID: 56479	*KCNQ5*	ID: 613605	5367–5413 (24)	−115÷−119	90–93
ID: 653319	*KIAA0895L*	ID: 512420	2878–2927 (25)	−115÷−119	90–93
ID: 11155	*LDB3*	ID: 536781	4420–450 (15)	−115÷−119	90–93
ID:10186	*LHFP*	ID: 532944	1396–1425 (14)	−115÷−119	90–93
ID: 108927	*NCDN*	ID: 505994	3556–3586 (16)	−115÷−119	90–93
ID: 7101	*NR2E1*	ID: 528156	2954–2980 (14)	−119	93
ID: 5579	*PRKCB*	ID: 282325	7060–7088 (15)	−115÷−121	90–95
ID: 862	*RUNX1T1*	ID: 538628	3268–3300 (17)	−115÷−119	90–93
ID: 388228	*SBK1*	ID: 614815	2320–2361 (20)	−117÷−121	92–95
ID: 23157	*SEPT6*	ID: 540783	4494–4522 (15)	−115÷−119	90–93
ID: 9342	*SNAP29*	ID: 532261	1360–1386 (14)	−115÷−119	90–93
ID: 54558	*SPATA6*	ID: 534169	3206–3232 (14)	−115÷−119	90–93
ID: 727837	*SSX2B*	ID: 534692	1276–1307 (16)	−115÷−121	90–95
ID: 10214	*SSX3*	ID: 6757	1085–1130 (18)	−115÷−121	90–95
ID: 11346	*SYNPO*	ID: 533531	3598–3624 (14)	−115÷−119	90–93
ID: 202500	*TCTE1*	ID: 523600	2239–2267 (15)	−115÷−119	90–93
ID: 84951	*TNS4*	ID: 532898	3472–3512 (21)	−115÷−119	90–93
ID: 79865	*TREML2*	ID: 515548	2346–2379 (17)	−115÷−119	90–93
ID: 134510	*UBLCP1*	ID: 508163	1544–1573 (15)	−115÷−119	90–93

### The Interaction of Bta-miRNAs and mRNA of Human Genes with High Complementarity

The total number of target genes for bta-miRNAs with 98–100% complementarity is 32 (Table 2). The nucleotide sequences of bta-miR-2881, bta-miR-2444, bta-miR-11975, bta-miR-135a, bta-miR-151-5p, bta-miR-1777b, bta-miR-1777a, bta-miR-2478, bta-miR-136, bta-miR-432, bta-miR-127, bta-miR-433, bta-miR-431 bta-miR-1282, and bta-miR-11976 BSs are full complementary to 13 mRNAs of human genes. The eight human miRNAs were identical to bovine miRNAs in name and nucleotide sequence. For these eight human miRNAs, the target genes indicated in Table 2 were experimentally verified (*Davis et al., 2005*; *Wang J. et al., 2016*; *Yurikova et al., 2019*).

**TABLE 2:** Characteristics of interactions of bta-miRNAs with human mRNA genes with high complementarity.

Gene	bta-miRNA	Start of site, nt	Region of miRNA	∆G, kJ/mole	∆G/∆Gm, %	Length, nt
*AR*	bta-miR-2881	416	5′UTR	−112	100	18
*CXorf38*	bta-miR-2444	1546	3′UTR	−93	100	20
*EGFR*	bta-miR-11975	87	5′UTR	−127	100	20
*GLYCTK*	bta-miR-135a^a^ ^,^ ^b^	2812	3′UTR	−113	100	23
*LPPR5*	bta-miR-151-5p^a^	1328	3′UTR	−113	100	21
*LYPD3*	bta-miR-151-5p^a^	1608	3′UTR	−113	100	21
*MEX3A*	bta-miR-1777b	301	CDS	−125	100	20
*MEX3A*	bta-miR-1777a	302	CDS	−123	100	20
*PTP4A2*	bta-miR-2444	2110	3′UTR	−93	100	20
*RBM43*	bta-miR-2478	2911	3′UTR	−106	100	20
*RHOB*	bta-miR-1777b	206	5′UTR	−125	100	20
*RTL1*	bta-miR-136^a^ ^,^ ^b^	110	CDS	−115	100	23
*RTL1*	bta-miR-432^a^ ^,^ ^b^	330	CDS	−123	100	23
*RTL1*	bta-miR-127^a^ ^,^ ^b^	1792	CDS	−121	100	22
*RTL1*	bta-miR-433^a^ ^,^ ^b^	2878	CDS	−119	100	22
*RTL1*	bta-miR-431^a^ ^,^ ^b^	3800	CDS	−127	100	23
*SERF2*	bta-miR-1282^a^	1072	CDS	−102	100	20
*ZIC5*	bta-miR-11976	1316	CDS	−134	100	21
*ZIC5*	bta-miR-11975	1317	CDS	−127	100	20
*ARID1A*	bta-miR-1584-5p	4587	CDS	−115	98	20
*ATP2B2*	bta-miR-2444	7003	3′UTR	−91	98	20
*BCAM*	bta-miR-6528	3012	3′UTR	−108	98	20
*CELF2*	bta-miR-2444	5313	3′UTR	−91	98	20
*CTSH*	bta-miR-2333	540	CDS	−117	98	21
*HCN2*	bta-miR-1777b	2374	CDS	−123	98	20
*HDX*	bta-miR-2444	5052	3′UTR	−91	98	20
*HOXB8*	bta-miR-196a^b^	1378	3′UTR	−110	98	22
*KLF9*	bta-miR-2897	799	5′UTR	−115	98	20
*MCRS1*	bta-miR-1584-5p	353	CDS	−115	98	20
*RFNG*	bta-miR-2412	1238	3′UTR	−125	98	22
*RGL2*	bta-miR-10173-5p	310	5′UTR	−117	98	22
*RHOB*	bta-miR-1777a	207	5′UTR	−121	98	20
*SEPT8*	bta-miR-151-3p^a^	2767	3′UTR	−110	98	21
*SLIT3*	bta-miR-1584-5p	57	5′UTR	−115	98	20
*SPAM1*	bta-miR-2285k	1320	CDS	−102	98	21
*TAF4*	bta-miR-1777b	763	CDS	−123	98	20
*TMEM164*	bta-miR-6528	2163	3′UTR	−108	98	20
*TNKS1BP1*	bta-miR-6528	5595	3′UTR	−108	98	20

a Identical miRNAs, with human.

b Milk miRNAs.

The mRNAs of 19 genes have BSs for 12 miRNAs with 98% complementarity. 15 mRNAs have BSs in 3′UTR, 10 mRNAs in CDS, and seven in 5′UTR and the free energy of interaction of miRNAs with mRNAs of these genes ranges from −93 to −127 kJ/mol. The bta-miR-2444 has five (*CXorf38, PTP4A2, ATP2B2, CELF2,* and *HDX)* human target genes, bta-miR-1777b (*MEX3A, RHOB,* and *HCN2*), bta-miR-6528 (*BCAM, TMEM164,* and *TNKS1BP1*), and bta-miR-1584-5p (*ARID1A, MCRS1,* and *SLIT3*) have three human target genes and bta-miR-151-5p (*LPPR5* and *LYPD3*), bta-miR-1777a (*MEX3A* and *RHOB*), and bta-miR-11975 (*EGFR* and *ZIC5*) has two human target genes. Other genes are targeted by one miRNA. Note that hsa-miR-619-5p binds fully complementary to the mRNA of more than 200 human genes (*Atambayeva et al., 2017*). The biological significance of the fully complementary binding of miRNAs to mRNAs remains a mystery since siRNAs typically destroy the mRNAs of the target gene.

The mRNA of the *MEX3A* gene has BSs for bta-miR-1777b and bta-miR-1777a. The mRNAs of *EGFR* and *RTL1* genes are targeted by bta-miR-11976 and bta-miR-431 with a high free energy of −127 kJ/mol. The gene targeted by the greatest number of miRNAs is *RTL1*, specifically five miRNAs: bta-miR-136, bta-miR-432, bta-miR-127, bta-miR-433, and bta-miR-431.

Despite the divergence of mammalian species, associations of miRNAs and their target genes persist for many millions of years, which indicate the importance of these associations in the regulation of genome expression. In recent years, quantitative characteristics of miRNA interactions with the mRNA of target genes have been established and the features of the organization of miRNA BS in mRNA of target genes have been revealed. This opens up new possibilities for regulating gene expression using miRNA (*Aisina et al., 2019*; *Mukushkina et al., 2020*; *Akimniyazova et al., 2021*; *Kamenova et al., 2021*). In particular, data on fully complementary miRNA and mRNA interactions raise questions about the function of such associations as they are similar to siRNA (Table 2). The complete and high complementarity of miRNA with the mRNA of many genes indicates a strong dependence of the participants in the miRNA and target gene associations.

**FIGURE 1:**
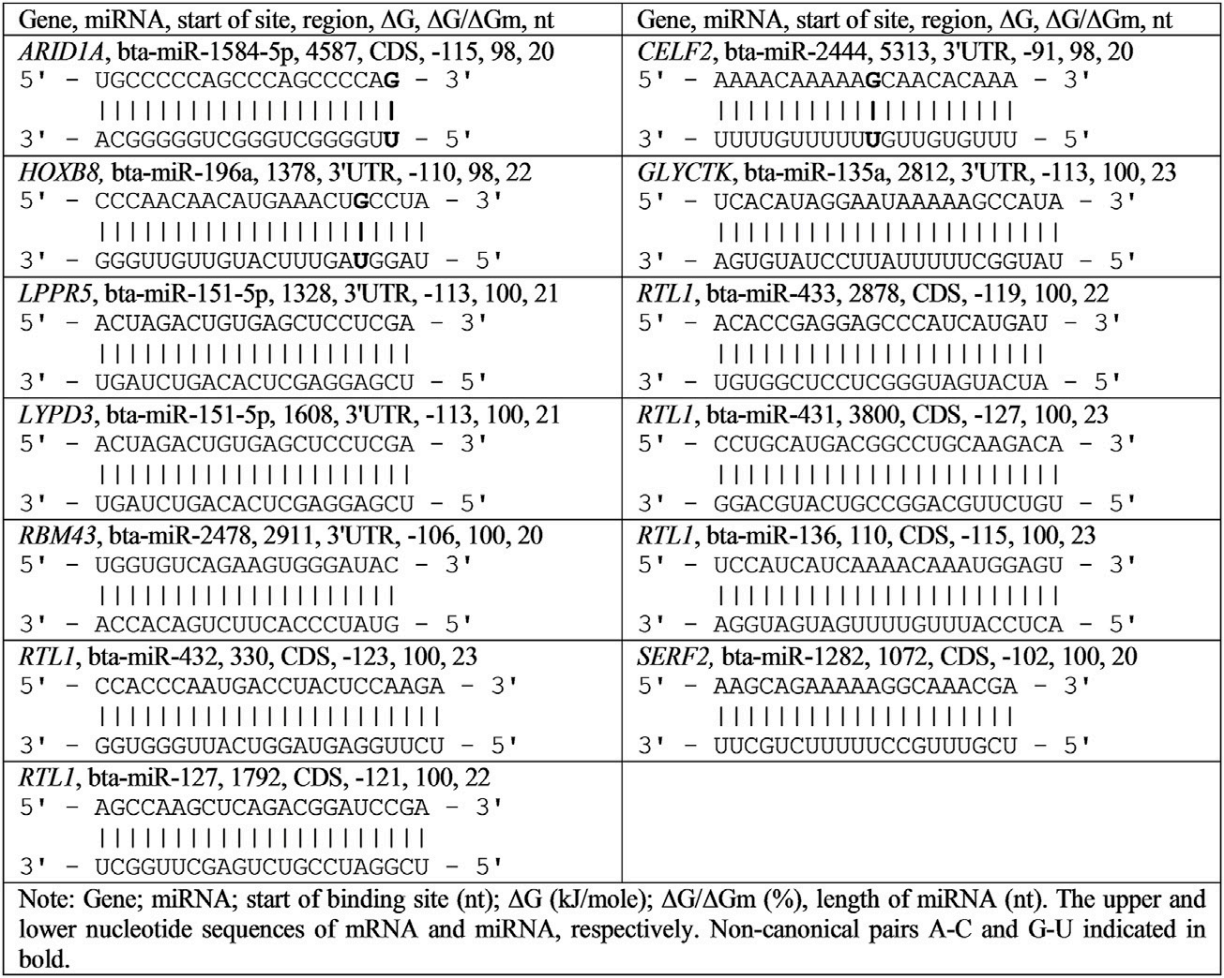
Schemes of the interaction of nucleotide sequences of bta-miRNAs and mRNA human genes.

The interaction of all nucleotides of miRNAs and mRNA of target genes shows how effectively these molecules bind. Figure 1 shows the construction of hydrogen bonds between all nucleotides of bta-miR-1584-5p, bta-miR-2444, and bta-miR-196a and their BSs in mRNA. Because the MirTarget program considers the interaction of the non-canonical pairs A-C and G-U, the interaction of miRNAs and mRNAs preserves the spiral structures of both molecules, and therefore, stacking interactions are found between all nucleotides of miRNA and mRNA, which stabilize the duplex (*Garg and Heinemann, 2018*).

### Characteristics of Bta-miR-11975, Bta-miR-11976, and Bta-miR-2885 Interactions with the CDS of mRNAs of Human Genes

As a result of the analysis of the interaction of 1025 bta-miRNAs with 17,508 human genes, we identified bta-miR-11975, bta-miR-11976, and bta-miR-2885, which had 118 human target genes. We predicted the cluster of BSs of bta-miR-11975, bta-miR-11976, and bta-miR-2885 in the mRNA of 66 human genes in CDSs. The BSs of these miRNAs (Table 3) consisted of repeating GCC triplets in CDS of mRNAs that encoded oligopeptides: polyP with a length of six amino acids (a.a.) in 16 genes, polyA six a.a. long in 17 genes, polyA seven a.a. long in eight genes, polyP seven a.a. long in four genes and polyR seven a.a. long in one gene, polyA eight a.a. long in five genes, polyP eight a.a. long in four genes, polyP nine a.a. long in one gene, polyP nine a.a. long in six genes, polyA ten a.a. long in two genes, polyA 11 a.a. long in one gene and polyP 12 a.a. long in one gene. There is a cluster 18 nt long of BSs of bta-miR-11975 and bta-miR-11976 in the 3′UTR of mRNA of only one human gene.

**TABLE 3:** Nucleotide sequences of BSs of bta-miR-11975, bta-miR-11976, and bta-miR-2885 in CDS mRNA of human genes.

Gene	18 nt length cluster	
*ATOH8*	5′-CUC​CCC​ACG​CCG​CCG​CCG​CCG​CCG​CCU​CCU​GCG​C-3′	PPPPPP
*GABBR2*	5′-CAG​CCC​GGG​CCG​CCG​CCG​CCG​CCG​CCA​CCG​CCG​C-3′	PPPPPP
*FAM117B*	5′-CCC​CCA​CGG​CCG​CCG​CCG​CCG​CCG​CCG​CUG​CUG​G-3′	PPPPPP
*FOXG1*	5′-CAG​CAG​CAG​CCG​CCG​CCG​CCG​CCG​CCC​CCG​GCA​C-3′	PPPPPP
*FOXK1*	5′-CCG​CCC​GGG​CCG​CCG​CCG​CCG​CCG​CCA​CCG​CCG​C-3′	PPPPPP
*GPR150*	5′-UCG​CCG​CUG​CCG​CCG​CCG​CCG​CCG​CCA​ACG​UCC​C-3′	PPPPPP
*IRX5*	5′-ACC​CCG​CGG​CCG​CCG​CCG​CCG​CCG​CCU​UCU​CCU​C-3′	PPPPPP
*LOXL1*	5′-CCC​UAC​GUG​CCG​CCG​CCG​CCG​CCG​CCC​CCC​GAC​G-3′	PPPPPP
*LTBP1*	5′-CUC​AGA​CCG​CCG​CCG​CCG​CCG​CCG​CCG​GAG​CCU​G-3′	RPPPPP
*MECP2*	5′-GGA​AAA​TGG​CCG​CCG​CCG​CCG​CCG​CCG​CGC​CGA​G-3′	PPPPPP
*TMEM121*	5′-AAC​UCG​GUG​CCG​CCG​CCG​CCG​CCG​CCG​CUG​CAC​G-3′	PPPPPP
*TSPYL2*	5′-CCG​CCC​CCG​CCG​CCG​CCG​CCG​CCG​CCG​CUC​CUC​C-3′	PPPPPP
*TPRN*	5′-CGC​CCC​CCG​CCG​CCG​CCG​CCG​CCG​CCC​GCG​CCG​C-3′	PPPPPP
*ZCCHC2*	5′-CGC​CCC​CCG​CCG​CCG​CCG​CCG​CCG​CCC​GCG​GGC​C-3′	PPPPPP
*ZNF367*	5′-GAG​AAC​CCG​CCG​CCG​CCG​CCG​CCG​CCC​GUC​AUC​U-3′	PPPPPP
*ZNF839*	5′-AAG​GCG​CAG​CCG​CCG​CCG​CCG​CCG​CCC​CCC​UUC​G-3′	PPPPPP
*FOXD1*	5′-CGC​AGC​GCG​CCG​CCG​CCG​CCG​CCG​CCU​UCC​ACC​C-3′	AAAAAA
*CASZ1*	5′-GCG​AGG​GCG​CCG​CCG​CCG​CCG​CCG​CCG​CAG​CUG​G-3′	AAAAAA
*GPR88*	5′-CCG​GCU​GCG​CCG​CCG​CCG​CCG​CCG​CCU​UCC​CGG​G-3′	AAAAAA
*FBXL17*	5′-UAU​CCU​CGG​CCG​CCG​CCG​CCG​CCG​CCG​CUG​CCG​C-3′	AAAAAA
*HOXA2*	5′-UUC​UGC​CGG​CCG​CCG​CCG​CCG​CCG​CCA​CCG​CCG​C-3′	AAAAAA
*HOXA13*	5′-CCG​CUG​CAG​CCG​CCG​CCG​CCG​CCG​CCG​CGU​CGU​C-3′	AAAAAA
*IRX2*	5′-CGG​CCG​ACG​CCG​CCG​CCG​CCG​CCG​CCG​GCU​UCC​C-3′	AAAAAA
*IRX3*	5′-UCU​CUC​CGG​CCG​CCG​CCG​CCG​CCG​CCG​CUC​ACA​G-3′	AAAAAA
*IRX4*	5′-CAG​CCA​CCG​CCG​CCG​CCG​CCG​CCG​CCA​CCU​CCC​U-3′	AAAAAA
*LCORL*	5′-CCG​CUG​CUG​CCG​CCG​CCG​CCG​CCG​CCG​CUC​AGU​G-3′	AAAAAA
*LHFPL3*	5′-CCG​CCG​CUG​CCG​CCG​CCG​CCG​CCG​CCG​CGA​UGC​U-3′	AAAAAA
*NANOS1*	5′-GCG​CGC​CCG​CCG​CCG​CCG​CCG​CCG​CCA​CCA​CCA​C-3′	AAAAAA
*POU3F3*	5′-UGC​CCC​ACG​CCG​CCG​CCG​CCG​CCG​CCG​CUG​CCG​C-3′	AAAAAA
*SOX12*	5′-AGG​GGG​CGG​CCG​CCG​CCG​CCG​CCG​CCU​CCC​CGA​C-3′	AAAAAA
*SOX21*	5′-CCG​CCG​CUG​CCG​CCG​CCG​CCG​CCG​CCG​CGG​GCA​G-3′	AAAAAA
*SP8*	5′-GCG​CCG​CAG​CCG​CCG​CCG​CCG​CCG​CCG​CAG​CCG​C-3′	AAAAAA
*UNCX*	5′-CUU​CCA​ACG​CCG​CCG​CCG​CCG​CCG​CCG​CGG​GGC​U-3′	AAAAAA
	21 nt length cluster	
*ARX*	5′-CGG​CCG​CUG​CCG​CCG​CCG​CCG​CCG​CCG​CCU​UCC​CGA​G-3′	AAAAAAA
*DGKI*	5′-CUC​CUG​CAG​CCG​CCG​CCG​CCG​CCG​CCG​CCA​GCC​CGC​C-3′	AAAAAAA
*GSG1L*	5′-CCG​CCC​CCG​CCG​CCG​CCG​CCG​CCG​CCG​CCA​CCG​CCU​C-3′	AAAAAAA
*JUND*	5′-CGG​CCG​CUG​CCG​CCG​CCG​CCG​CCG​CCG​CCG​GGG​GGC​C-3′	AAAAAAA
*SKOR2*	5′-CCG​GCC​CCG​CCG​CCG​CCG​CCG​CCG​CCG​CCC​CCG​CCG​C-3′	PPPPPPP
*CEBPA*	5′-CCU​UAC​CAG​CCG​CCG​CCG​CCG​CCG​CCG​CCC​UCG​CAC​C-3′	PPPPPPP
*CHD3*	5′-CUC​UUC​CCG​CCG​CCG​CCG​CCG​CCG​CCG​CCA​CCG​CUG​C-3′	PPPPPPP
*CTNND2*	5′-GAG​CCC​GCG​CCG​CCG​CCG​CCG​CCG​CCG​CCG​CGG​GAG​C-3′	PPPPPPP
*HCN2*	5′-GCG​CCG​GGG​CCG​CCG​CCG​CCG​CCG​CCG​CCC​GCG​CCC​C-3′	PPPPPPP
*HTT*	5′-CCG​CCA​CCG​CCG​CCG​CCG​CCG​CCG​CCG​CCU​CCU​CAG​C-3′	PPPPPPP
*SOBP*	5′-CCC​GAG​CAG​CCG​CCG​CCG​CCG​CCG​CCG​CCC​GCG​CCC​C-3′	PPPPPPP
*TGFBR3L*	5′-CCU​CUG​ACG​CCG​CCG​CCG​CCG​CCG​CCG​CCA​UCG​CGG​U-3′	PPPPPPP
*SLC24A3*	5′-CGC​GCG​UCG​CCG​CCG​CCG​CCG​CCG​CCG​CCG​GAG​GGA​C-3′	RRRRRRR
	24 nt length cluster	
*CCDC177*	5′-GCC​CCG​CGG​CCG​CCG​CCG​CCG​CCG​CCG​CCG​CCG​CGG​CCU​C-3′	AAAAAAAA
*IRS2*	5′-AGC​CCA​GGG​CCG​CCG​CCG​CCG​CCG​CCG​CCG​CCG​UGC​CUU​C-3′	AAAAAAAA
*MEGF9*	5′-UGU​GCU​GCG​CCG​CCG​CCG​CCG​CCG​CCG​CCG​CCG​UCG​CCU​C-3′	AAAAAAAA
*SKIDA1*	5′-ACC​CGG​CAG​CCG​CCG​CCG​CCG​CCG​CCG​CCG​CCG​CUG​CUG​C-3′	AAAAAAAA
*ZIC3*	5′-CAA​CCC​ACG​CCG​CCG​CCG​CCG​CCG​CCG​CCG​CCG​CUG​CCU​U-3′	AAAAAAAA
*FMNL1*	5′-GUG​CCU​CCG​CCG​CCG​CCG​CCG​CCG​CCG​CCG​CCU​CCC​GGA​G-3′	PPPPPPPP
*GBX2*	5′-GUA​GUG​CUG​CCG​CCG​CCG​CCG​CCG​CCG​CCG​CCC​GCG​CUG​C-3′	PPPPPPPP
*MMP24*	5′-GCG​CCG​GGG​CCG​CCG​CCG​CCG​CCG​CCG​CCG​CCG​GGC​CAG​G-3′	PPPPPPPP
*TRIM67*	5′-CUG​GUG​CAG​CCG​CCG​CCG​CCG​CCG​CCG​CCG​CCC​GCC​GAG​G-3′	PPPPPPPP
	27–36 nt length cluster	
*DLX6*	5′-CCT​GCC​CGG​CCG​CCG​CCG​CCG​CCG​CCG​CCG​CCG​CCG​CAG​CCG​CCT​CGC​AGC​A-3′	PPPPPPPPP
*DMRTA2*	5′-GCG​TCG​ACG​CCG​CCG​CCG​CCG​CCG​CCG​CCG​CCG​CCG​GGG​GGC​CTG​GGC​TGC​C-3′	AAAAAAAAA
*FOXF2*	5′-CGC​CGC​CCG​CCG​CCG​CCG​CCG​CCG​CCG​CCG​CCG​CCC​CGG​AGA​CCA​CCT​CCT​C-3′	AAAAAAAAA
*IRF2BPL*	5′-TAA​GCG​CTG​CCG​CCG​CCG​CCG​CCG​CCG​CCG​CCG​CCG​CTG​CGG​TGG​AAC​AGC​G-3′	AAAAAAAAA
*MNX1*	5′-CGG​CCG​CTG​CCG​CCG​CCG​CCG​CCG​CCG​CCG​CCG​CCG​CTG​GGG​GCC​TGG​CGC​T-3′	AAAAAAAAA
*NKX2-3*	5′-CGG​CCG​CGG​CCG​CCG​CCG​CCG​CCG​CCG​CCG​CCG​CCG​CAG​CAG​CGG​CGG​CCT​A-3′	AAAAAAAAA
*ZNF703*	5′-TGG​GCA​GCG​CCG​CCG​CCG​CCG​CCG​CCG​CCG​CCG​CCT​CCT​GCC​ATC​TGC​ACC​T-3′	AAAAAAAAA
*ZSWIM6*	5′-CCG​CCG​CTG​CCG​CCG​CCG​CCG​CCG​CCG​CCG​CCG​CCG​CCG​CGG​GGG​CCG​GGG​C-3′	AAAAAAAAAA
*CASKIN1*	5′-CCC​CGC​GAG​CCG​CCG​CCG​CCG​CCG​CCG​CCG​CCG​CCG​CCG​CGC​CCC​CCG​CCC​C-3′	AAAAAAAAAA
*FOXE1*	5′-GCT​GCC​CAG​CCG​CCG​CCG​CCG​CCG​CCG​CCG​CCG​CCG​CCG​CCA​TCT​TCC​CAG​G-3′	AAAAAAAAAAA
*ZIC5*	5′-GCC​GGG​CTG​CCG​CCG​CCG​CCG​CCG​CCG​CCG​CCG​CCG​CCG​CCG​CCA​CCG​CCC​C-3′	PPPPPPPPPPP

**FIGURE 2:**
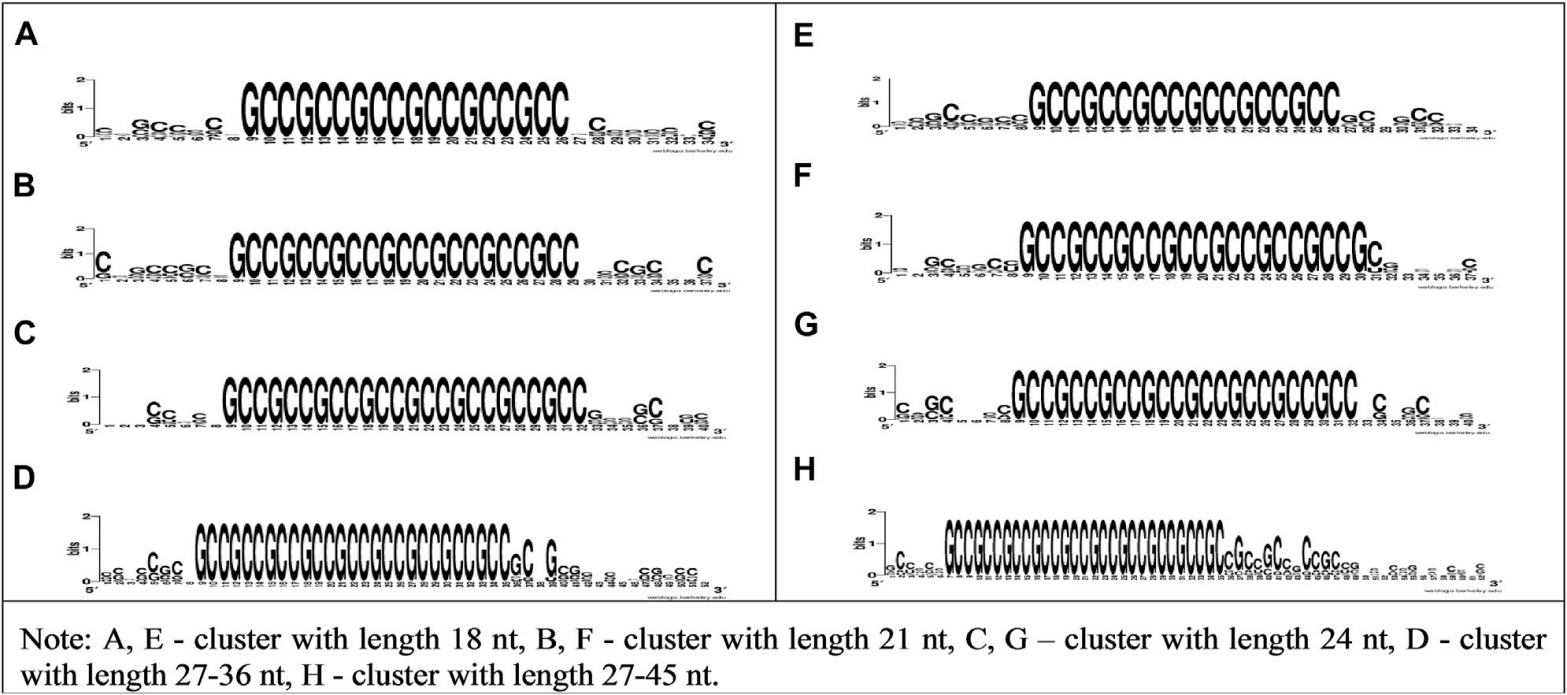
WebLogo schemes of nucleotide sequences variability of CDS and 5′UTR mRNA having bta-miR-11976, bta-miR-11975, and bta-miR-2885 BSs clusters with lengths of 18, 21, 24, 27–36, and 27–45 nt.

Figure 2 shows the high conservatism of bovine miRNAs BSs in CDS and 5′UTR mRNAs of human genes. To confirm the interaction of cluster of bta-miR-11975, bta-miR-11976, and bta-miR-2885 BSs with human genes, we plotted the WebLogo for mRNA sections containing conservative BSs in CDS and 5′UTR with various lengths, specifically 18, 21, 24, 27–36, and 27–45 nt.

Supplementary Tables S3–S6 show characteristics of the interaction of bta-miR-11975, bta-miR-11976, and bta-miR-2885 BSs with the mRNA of human genes in a cluster. The mRNAs of *CASZ1, FOXK1, NANOS1, POU3F3,* and *TSPYL2* genes have clusters of BSs for three miRNAs, and other genes can bind two miRNAs. The mRNAs of *GPR88, LOXL1, LTBP1, MECP2,* and *TMEM121* genes can bind single bta-miR-11975, with a free energy −115 to −117 kJ/mol. BSs of bta-miR-11976 and bta-miR-11975 in mRNAs of *ATOH8, GPR150, FAM117B, FOXD1, HOXA2, TPRN, ZNF367,* and *ZNF839* genes are located through one nucleotide. In mRNAs of *CASZ1, NANOS1, POU3F3, TSPYL2,* and *UNCX* genes BSs of bta-miR-11976 and bta-miR-2885 are located with overlapping, that is, they form a cluster. The mRNAs of *FOXG1, LCORL,* and *SOX12* genes have multiple BSs for bta-miR-11975 located through three nucleotides. Therefore, of these three miRNAs, only one can bind to the mRNA of target genes. At equal concentrations of miRNAs, it will preferentially bind with bta-miR-11976 since it has more free energy of interaction with the mRNA. However, at significantly higher concentrations of bta-miR-11975 and bta-miR-2885, they will preferentially bind to the mRNA of the target gene.

The mRNA of the *GABBR2* gene is characterized by the presence of two clusters: the first cluster starts at 276 nt and ends at 306 nt, with a length of 30 nt. The second cluster is 32 nt long, starting at 487 nt and ending at 519 nt. The bta-miR-11976 interacts with *HOXA13* mRNA with the free energy more than −129 kJ/mol and has two BSs: the first starts at 399 nt and ends at 428 nt, and the second starts at 600 nt and ends at 641 nt. The mRNA of the *FBXL17* gene contains four BSs for bta-miR-11975 and two BSs for bta-miR-11976 located through three nucleotides. The mRNAs of *IRX2* and *IRX4* genes have three BSs for bta-miR-11975 and two BSs for miR-11976 located through three nucleotides.

The mRNA of the *POU3F3* gene contains multiple BSs for three miRNAs which present in two clusters. The cluster size is 41 nt. The first cluster starts at position 304 nt and ends at position 345 nt. The mir-11975 has eight BSs and miR-11976 has six BSs located through three nucleotides. The free energy of interactions of the three miRNAs with the mRNAs ranges from −110 to −123 kJ/mol. The second cluster starts at position 583 to 612 nt, and the cluster size is 29 nt. The free energy of interactions ranges from −110 to −121 kJ/mol. It is noteworthy that *POU3F3* is characterized by the presence of two clusters and many BSs when compared to other target genes. This suggests that this gene is more susceptible to regulation by miRNA.

The mRNA of the *SP8* gene contains five BSs for bta-miR-11976 and six BSs bta-miR-11975 located through three nucleotides. The cluster starts at 553 nt and ends at 590 nt, with a length of 37 nt and ∆G/∆G_m_ of 92%. The free energy of interactions of two miRNAs with the mRNA varied from −117 to −123 kJ/mol. The mRNA of *LHFPL3* and *SOX21* genes have five BSs for bta-miR-11975 and three for bta-miR-11976 with a length of 32 and 37 nt, respectively.

Supplementary Table S4 shows interactions of mRNAs of 13 human genes with bta-miRNAs with a length of 21 nt. In all mRNAs, bta-miR-11975 and bta-miR-11976 are located through one nucleotide. The mRNAs of *ARX, CHD3, DGK1,* and *JUND* genes contain three BSs for bta-miR-11975 and two BSs for bta-miR-11976. In the mRNA of the *JUND* gene, bta-miR-11976 and bta-miR-2885 BSs are located through three nucleotides.

The mRNAs of *CTNND2* and *HTT* genes are characterized by clusters for three miRNAs BSs: bta-miR-11975, bta-miR-11976, and bta-miR-2885. Each of the mRNAs has four BSs for bta-miR-11975, three BSs for bta-miR-11976, and two BSs for bta-miR-2885 located through six nucleotides. The miR-2885 and bta-miR-11976 BSs are located with overlapping.

The mRNA of the *GSGL* gene has six BSs for bta-miR-11976 and bta-miR-11975. The mRNA of the *HCN2* gene has two BSs for bta-miR-11976 and bta-miR-11975 located from 109 to 136 nt, and the other from 152 to 175 nt, respectively. The cluster of BSs in mRNA of the *SKOR2* gene is one of the largest. The first cluster ranges from 851 to 882 nt, with a length of 30 nt. The second cluster starts at 2078 nt and ends at 3014 nt, with a length of 36 nt. In the second cluster, bta-miR-11976 and bta-miR-2885 BSs are located through six nucleotides. The mRNAs of *SLC24A3* and *TGFBR3L* genes have a cluster consisting of multiple BSs for bta-miR-11975 and bta-miR-11976. The mRNA of the *SOBP* gene has bta-miR-1975 BSs located through three nucleotides and overlapping with nucleotide sequences of bta-miR-11976 BSs. The bta-miRNA-11976 can interact with the mRNAs of *CHD3, CTNND2, GSG1L, HTT,* and *SKOR2* genes with a free energy of more than −120 kJ/mol.

Supplementary Table S5 shows characteristics of interactions of bta-miRNAs with mRNAs of nine human genes in the cluster with a length of 24 nt. The mRNAs of *CCDC177, GBX2,* and *TRIM67* genes have BSs for bta-miR-11975 and bta-miR-11976. The mRNA of the *MMP4* gene has two BSs for bta-miR-11975 and bta-miR-2885. The free energy of interaction value is equal to −127 kJ/mol.

The mRNAs of the *FNML1, IRS2, MEGF9,* and *ZIC3* genes have a cluster of multiple BSs for bta-miR-11975, bta-miR-11976, and bta-miR-2885, BSs are located through three nucleotides. The mRNA of the *SKIDA1* gene contains a cluster for three miRNAs BSs with a total length of 68 nt, starting at 2917 nt and ending 2985 nt.

Supplementary Table S6 shows the characteristics of mRNA of 11 human genes with bta-miRNAs in the clusters with a length of 27–36 nt. The mRNA of the *DLX6* gene has multiple BSs for bta-miR-11975 and bta-miR-11976. The mRNAs of 10 genes contains clusters for bta-miR-11975 and bta-miR-11976 and bta-miR-2885. In all mRNAs of 10 genes BSs of bta-miR-11976 and bta-miR-2885 are located with overlapping.

The mRNA of the *DMRTA2* gene contains four BSs for bta-miR-11975, bta-miR-11976, and bta-miR-2885. The mRNA of the *FOXF2* gene contains three BSs for these miRNAs. The mRNA of the *IRF2BPL* gene contains five BSs for bta-miR-11975 and bta-miR-11976 and three BSs for bta-miR-2885. The mRNA of the *MNX1* gene has a cluster of BSs for bta-miR-11975, bta-miR-11976, and bta-miR-2885. The cluster size is 45 nt and ∆G varied from −110 to −127 kJ/mol. The mRNA of the *NKX2-3* gene contains a single BS for bta-miR-11976, six BSs for bta-miR-11975, and three BSs for bta-miR-2885. The mRNA of the *ZNF703* gene contains a cluster for three miRNA BSs, the length of the cluster is 31 nt, extending from 1711 to 1742 nt. The mRNAs of *CASKIN1, FOXE1,* and *ZSWIM6* genes contain a cluster consisting of multiple BSs for bta-miR-11976, bta-miR-11975, and bta-miR-2885. The mRNA of the *ZIC5* gene is characterized by a single BS for bta-miR-2885 and bta-miR-11976 and two BSs for bta-miR-11976 located through six nucleotides. The second cluster has 10 BSs for bta-miR-11976, eight BSs for bta-miR-11976, and five BSs for bta-miR-2885, extending from 1467 nt to 1517 nt, the length is 50 nt, and ∆G value ranges from −110 to −127 kJ/mol.

Characteristics of bta-miR-11975, bta-miR-11976, and bta-miR-2885 interactions with the 5′UTR of mRNAs of human genes.

Clusters of BSs for bta-miR-11975, bta-miR-11976, and bta-miR-2885 in 5′UTR mRNA were identified in 52 human genes. Table 4 shows nucleotide sequences of bta-miRNAs BSs in 5′UTR mRNA of human genes formed from GCC trinucleotides clusters with a length of 18 nt in mRNA of 18 genes, with a length of 21 nt in mRNA of 11 genes, with a length of 24 nt in mRNA of 14 genes, with a length of 27 nt in mRNA of two genes, with a length of 30 nt in mRNA of two genes, with a length of 36 nt in mRNA of two genes, and with a length of 39, 42, and 48 nt in the mRNA of one gene.

**TABLE 4:** Nucleotide sequences of BSs of bta-miR-11975, bta-miR-11976, and bta-miR-2885 in 5′UTR human mRNAs.

Gene	18 nt length cluster
*ABCC1*	5′-CUC​CCU​GCG​CCG​CCG​CCG​CCG​CCG​CCG​CAG​CGC​U-3′
*ASH1L*	5′-CUG​CUG​CUG​CCG​CCG​CCG​CCG​CCG​CCG​CUC​CCG​C-3′
*BTF3L4*	5′-CUG​CUC​CCG​CCG​CCG​CCG​CCG​CCG​CCG​UCG​UCU​U-3′
*C2CD4C*	5′-ACU​GCG​CUG​CCG​CCG​CCG​CCG​CCG​CCC​GCA​UCG​A-3′
*CPT1A*	5′-ACU​CCA​CCG​CCG​CCG​CCG​CCG​CCG​CCG​CUG​CCG​C-3′
*EGLN1*	5′-UCG​CCG​UCG​CCG​CCG​CCG​CCG​CCG​CCA​UGG​CCA​A-3′
*GRIN1*	5′-UCC​GCG​GAG​CCG​CCG​CCG​CCG​CCG​CCG​GGC​CCU​U-3′
*GTF2E2*	5′-CCG​CCG​CUG​CCG​CCG​CCG​CCG​CCG​CCA​CCG​CCA​G-3′
*MAST1*	5′-CUC​CCC​GCG​CCG​CCG​CCG​CCG​CCG​CCU​CCG​CCG​C-3′
*MEMO1*	5′-CCG​CUC​CUG​CCG​CCG​CCG​CCG​CCG​CCU​CCU​CAU​U-3′
*MPRIP*	5′-AGG​CCU​GCG​CCG​CCG​CCG​CCG​CCG​CCG​UCG​CCG​C-3′
*NOG*	5′-GCG​CGG​ACG​CCG​CCG​CCG​CCG​CCG​CCG​CUG​GAG​U-3′
*RIMS4*	5′-AGC​CGC​CCG​CCG​CCG​CCG​CCG​CCG​CCG​CGG​CCG​A-3′
*RNF165*	5′-CGC​GCG​CAG​CCG​CCG​CCG​CCG​CCG​CCG​CGC​GAG​G-3′
*RNF220*	5′-CUG​CCG​CUG​CCG​CCG​CCG​CCG​CCG​CCG​CUG​CCU​C-3′
*SCAP*	5′-CCC​CCG​UCG​CCG​CCG​CCG​CCG​CCG​CCG​CAG​CUU​G-3′
*SEPHS1*	5′-GGG​CCC​CCG​CCG​CCG​CCG​CCG​CCG​CCG​GGC​GCG​G-3′
*SPEN*	5′-CCG​CCG​CAG​CCG​CCG​CCG​CCG​CCG​CCC​CGG​CAC​C-3′
	21 nt length cluster
*ABCD3*	5′-GTA​AGG​UAG​CCG​CCG​CCG​CCG​CCG​CCG​CCG​CGU​CCC​C-3′
*ANKH*	5′-AAC​CUU​CUG​CCG​CCG​CCG​CCG​CCG​CCG​CCG​UCC​CTC​C-3′
*ANKRD13D*	5′-GCC​CCG​CUG​CCG​CCG​CCG​CCG​CCG​CCG​CCG​CUA​CTG​C-3′
*C4orf19*	5′-GGG​ACC​CCG​CCG​CCG​CCG​CCG​CCG​CCG​CCG​UCU​GGC​C-3′
*CA10*	5′-UGG​CUG​CUG​CCG​CCG​CCG​CCG​CCG​CCG​CCG​CUG​CTA​G-3′
*DISP2*	5′-CCG​CCA​CCG​CCG​CCG​CCG​CCG​CCG​CCG​CCG​CGG​CTT​C-3′
*HS3ST4*	5′-CGG​GGG​CUG​CCG​CCG​CCG​CCG​CCG​CCG​CCG​CGA​GCC​G-3′
*JARID2*	5′-GUG​GUG​CUG​CCG​CCG​CCG​CCG​CCG​CCG​CCG​CUG​GAG​T-3′
*RGP1*	5′-CAG​CGG​ACG​CCG​CCG​CCG​CCG​CCG​CCG​CCG​CGU​ACC​T-3′
*UBE2R2*	5′-GGC​CCG​GCG​CCG​CCG​CCG​CCG​CCG​CCG​CCG​CGA​UGG​C-3′
*USP25*	5′-GCG​CCA​CCG​CCG​CCG​CCG​CCG​CCG​CCG​CCG​CGG​GGG​C-3′
	24 nt length cluster
*AFF2*	5′-CAG​CCG​CUG​CCG​CCG​CCG​CCG​CCG​CCG​CCG​CCG​CGC​CGC​C-3′
*CUL3*	5′-GAG​UCC​GAG​CCG​CCG​CCG​CCG​CCG​CCG​CCG​CCC​CCG​CCG​C-3′
*FAM50A*	5′-CGC​CGC​CCG​CCG​CCG​CCG​CCG​CCG​CCG​CCG​CCG​CUG​CCA​U-3′
*GSK3B*	5′-GGG​CUU​GUG​CCG​CCG​CCG​CCG​CCG​CCG​CCG​CCC​GGG​CCA​A-3′
*MAP2K3*	5′-CCG​CAG​UCG​CCG​CCG​CCG​CCG​CCG​CCG​CCG​CCG​CUG​CUC​C-3′
*MSI1*	5′-CGC​CGA​GCG​CCG​CCG​CCG​CCG​CCG​CCG​CCG​CCG​CUC​CGC​U-3′
*MTHFD1L*	5′-UCC​UUC​CCG​CCG​CCG​CCG​CCG​CCG​CCG​CCG​CCU​GCU​CCC​C-3′
*NCKAP1*	5′-CCG​GAG​ACG​CCG​CCG​CCG​CCG​CCG​CCG​CCG​CCA​CAC​CUA​G-3′
*RPRD2*	5′-CCG​CUC​CCG​CCG​CCG​CCG​CCG​CCG​CCG​CCG​CCA​GAG​GAG​C-3′
*UBTF*	5′-CAG​CCA​CAG​CCG​CCG​CCG​CCG​CCG​CCG​CCG​CCA​CAG​CAG​C-3′
*TCEA1*	5′-GAG​CCG​GAG​CCG​CCG​CCG​CCG​CCG​CCG​CCG​CCG​CGG​GCU​U-3′
*THOC7*	5′-CAG​CUU​GCG​CCG​CCG​CCG​CCG​CCG​CCG​CCG​CCG​CGC​ACG​C-3′
*USP7*	5′-GGC​CGC​CCG​CCG​CCG​CCG​CCG​CCG​CCG​CCG​CCC​CGG​CUC​G-3′
*ZNF219*	5′-CGC​CGC​CCG​CCG​CCG​CCG​CCG​CCG​CCG​CCG​CCC​GCU​CCG​C-3′
	27–48 nt length cluster
*SBF1*	5′-ACC​UGG​GCC​GCC​GCC​GCC​GCC​GCC​GCC​GCC​GCC​GCG​GAG​CGA​ACC​AGG​GGU​GUC​CGG​GGT-3′
*SMAD9*	5′-GCU​GGG​GCC​GCC​GCC​GCC​GCC​GCC​GCC​GCC​GCC​GCU​GCU​GCA​GCC​GCU​GUC​UCG​GUC​CCC-3′
*BCL11A*	5′-CCG​CCC​GCC​GCC​GCC​GCC​GCC​GCC​GCC​GCC​GCC​GCC​CGC​CCC​GCA​GCC​CAC​CAU​GUC​TCG-3′
*WBP4*	5′-GCU​GCU​GCC​GCC​GCC​GCC​GCC​GCC​GCC​GCC​GCC​GCC​GCU​GCU​GCU​GCC​CAC​ACG​CUC​CCG-3′
*GNB2*	5′-AUC​CGC​GCC​GCC​GCC​GCC​GCC​GCC​GCC​GCC​GCC​GCC​GCC​GCC​UCC​GCC​GCG​GAG​GAA​GAC-3′
*KIF3B*	5′-GCC​CCC​GCC​GCC​GCC​GCC​GCC​GCC​GCC​GCC​GCC​GCC​GCC​GCC​CGC​UUU​CGG​CUC​GGG​CCT-3′
*NDRG3*	5′-CCU​CUC​GCC​GCC​GCC​GCC​GCC​GCC​GCC​GCC​GCC​GCC​GCC​GCC​GCC​GCU​GCU​GCU​GCA​CTG-3′
*BCL2L11*	5′-GCC​GCU​GCC​GCC​GCC​GCC​GCC​GCC​GCC​GCC​GCC​GCC​GCC​GCC​GCC​GCC​ACU​ACC​ACC​ACT-3′
*RHOT1*	5′-GAC​UCG​GCC​GCC​GCC​GCC​GCC​GCC​GCC​GCC​GCC​GCC​GCC​GCC​GCC​GCC​GCC​GCC​ACA​GCC-3′

Supplementary Tables S7–S10 show characteristics of bta-miR-11975, bta-miR-11976, and bta-miR-2885 BSs in mRNAs of human genes located in a cluster. The *ABCC1* mRNA is predicted to be targeted by three cow miRNAs. Three BSs were identified for bta-miR-11975, two BSs for bta-miR-11976, and one BS for bta-miR-2885. The BSs of bta-miR-11976 and bta-miR-2885 overlap. The mRNA of the *ASH1L* gene has two BSs for bta-miR-11975. The mRNA of the *BTF3L4* gene has two BSs for bta-miR-11975 and bta-miR-11976 located through six nucleotides. The mRNA of the *C2CD4C* gene interacts with a single miRNA.

BSs of bta-miR-11975 and bta-miR-11976 were identified in mRNAs of *CPT1A*, *GTF2E2,* and *MAST1* genes. BSs of bta-miR-11975 and bta-miR-11976 are located through one nucleotide. Bta-miR-11976 has four multiple BSs, bta-miR-11975 has five, four and three BSs in mRNAs of target genes, respectively. The mRNAs of *EGLN1* and *RIMS4* have three BSs for bta-miR-11975 and two BSs for bta-miR-11976. The mRNAs of *GRIN1* and *MEMO1* have single BSs for bta-miR-11975 and bta-miR-11976.

BSs of bta-miR-11975 and bta-miR-11976 are identified in mRNAs of *MPRIP, RNF165,* and *RNF220* genes, located through one nucleotide. In mRNAs of *NOG, SCAP, SEPHS1,* and *SPEN* genes identified clusters for BSs of bta-miR-11975, bta-miR-11976 and bta-miR-2885. In mRNAs of *NOG, SCAP,* and *SPEN* genes BSs of bta-miR-11976 and bta-miR-2885 are overlapping.

Supplementary Table S8 shows interactions of bta-miRNAs BSs in a cluster with a length of 21 nt in mRNAs of 11 human genes. The mRNAs of *ABCD3, C4orf19, DISP2, HS3ST4, RGP1, UBE2R2,* and *USP25* genes have clusters of multiple BSs for bta-miR-11975, bta-miR-11976, and bta-miR-2885. BSs of bta-miR-11976 and bta-miR-2885 in clusters are located with overlapping through three nucleotides.

The mRNAs of *ANKH, ANKRD13D,* and *CA10* genes have BSs for bta-miR-11975 and for bta-miR-11976. The mRNA of *ANKRD13D* gene contains five BSs for bta-miR-11975 and four BSs for bta-miR-11976 located through three nucleotides. The mRNAs of *CA10* and *JARID* genes has three BSs for bta-miR-11975 and mRNA of *ANKH* gene has two BSs. The mRNAs of *ANKH*, *CA10,* and *JARID* genes have two BSs for bta-miR-11976. The BSs for bta-miR-11975 and bta-miR-11976 are located through one nucleotide. The free energy of interactions ∆G ranges from −110 to −127 kJ/mol.

The cluster in mRNA of the *UBE2R2* gene starts at 541 nt and ends at 569 nt, with a length of 28 nt. The total BSs length in the cluster is 281 nt, where the degree of compaction is 10.

BSs of mRNAs of 14 human genes contain a cluster with a length of 24 nt for bta-miRNAs (Supplementary Table S9). The free energy of interactions between miRNAs and the mRNAs of genes varied from −110 to −127 kJ/mol. The mRNA of the *AFF2* gene contains one cluster with two BSs for bta-miR-11975 and bta-miR-11976. The cluster starts at 14 nt and ends at 73 nt, with a length of 59 nt. The total length of BSs in the cluster is 724 nt. BSs of bta-miR-11975 and bta-miR-11976 are located through three nucleotides, starting at 101 nt and ending at 131 nt.

The mRNAs of *CUL3, FAM50A, MAP2K3, MSI1, MTHFD1L, NCKAP1, RPRD2, TCEA1, THOC7,* and *ZNF219* genes have a cluster of BSs for bta-miR-11975, bta-miR-11976, and bta-miR-2885. BSs of bta-miR-11976 and bta-miR-2885 are located with overlapping. The mRNA of *CUL3* has six BSs for bta-miR-11975 and bta-miR-11976. Bta-miR-2885 has a single BS located at 200 nt. The mRNAs of *FAM50A* and *MAP2K3* genes have five BSs for bta-miR-11975, four BSs for bta-miR-11976, and three BSs for bta-miR-2885. The mRNAs of *MSI1*and *RPRD2* genes contain four BSs for bta-miR-11975, three BSs for bta-miR-11976, and bta-miR-2885. The mRNAs of *MTHFD1L* and *NCKAP1* genes have four and three bta-miR-11975 BSs, respectively. The BSs of bta-miR-11976 and bta-miR-11975 are located with overlapping. The *ZNF219* mRNA has a cluster of BSs for bta-miR-11975, bta-miR-11976, and bta-miR-2885. The mRNAs of *TCEA1* and *THOC7* have three BSs for bta-miR-11975 and bta-miR-11976, and two BSs for bta-miR-2885.

The mRNAs of *GSK3B* and *UBTF* contain BSs for bta-miR-11975 and bta-miR-11976. The mRNA of the *USP7* gene has a single BS for bta-miR-11975 and bta-miR-11976 and cluster of BSs for three miRNAs. The cluster of BSs for bta-miR-11975, bta-miR-11976, and bta-miR-2885 is located from 94 to 121 nt, 27 nt.

Supplementary Table S10 shows characteristics of interactions of bta-miR-11975, bta-miR-11976, and bta-miR-2885 with nine genes mRNAs containing the BSs clusters with a length of 27–45 nt. The mRNAs of *SBF1* and *SMAD9* genes have four BSs for bta-miR-11975 and bta-miR-11976 and three BSs for bta-miR-2885. The mRNAs of *BCL11A, BCL2L11,* and *GNB2* genes have multiple BSs for bta-miR-11975 and bta-miR-11976. The BSs clusters for three miRNAs were identified in mRNA of *WBP4* gene. The mRNA of the *KIF3B* gene is a target for three miRNAs and the total length of BSs is 94 nt. The mRNA of the *NDRG3* gene is a target for three miRNAs, two of which have nine BSs. The mRNA of *RHOT1* has a cluster of BSs for bta-miR-11975, bta-miR-11976, and bta-miR-2885. The cluster size is 47 nt and the total BS length in the cluster is 560 nt. The free energy value is higher than −127 kJ/mol for the interactions of bta-miR-11976 with the mRNAs of all nine human genes.

### The miRNAs with Multiple Targets Have Been Shown to Have Several Effects on Various Diseases in Humans, Including Cancer

Supplementary Table S11 shows data for genes targeted by bta-miRNAs that may be involved in the development of various diseases: six genes associated with breast cancer; *CTNND2* – liposarcoma; *CEBPA -* myeloid leukemia; *DGKI -* gastric cancer; *FBXL17 -* medulloblastoma; *FMNL1*, *FOXD1,* and *SKOR2* – cell carcinoma; *FOXE1 -* thyroid cancer; *FOXK1, IRS2* cholangiocarcinoma; *IRX3 -* hepatocellular carcinoma; *MNX1, SOX12, TRIM67,* and *ZNF839* - colorectal cancer; *HOXA2* and *HOXA13 -* prostate cancer; *LOXL1 -* pancreatic cancer; *DLX6, SOBP,* and *UNCX -* lung cancer; *LHFPL3 -* melanoma; *LTBP1 -* glioblastoma; *SOX21 -* cervical cancer; *SP8 -* hepatoblastoma; *SLC24A3* – meningiomas; *TGFBR3L -* neuroendocrine tumors; *TPRN–*myeloma; *ZCCHC2 -* retinoblastoma; *ZNF703 -* thyroid carcinoma and neurodegenerative diseases: *ARX -* interneuron development; *CCDC177, CHD3, TSPYL2, FOXG1,* and *ZSWIM6 -* neurodevelopmental syndrome; *DMRTA2 -* cortical development; *GABBR2 -* autism; *GPR88* and *POU3F3 -* Parkinson’s disease; *HCN2 -* epilepsy; *HTT -* Huntington’s disease; *IRF2BPL -* neurological phenotypes; *LCORL* and *MMP24 -* Alzheimer’s disease*; MECP2 -* neurodevelopmental disorder; *CASZ1, GBX2, IRX4, IRX5, NKX2-3, JUND,* and *ZIC3 -* cardiac diseases; *GPR150 -* liver disease. This list of candidate genes for various human diseases indicates the great potential of bta-miRNAs in the regulation of the pathogenesis of the listed diseases. Increasing evidence sheds light on the potential implications of ex-miRs identified in human biofluids derived from non-human species to cross-kingdom gene regulation and human disease pathogenesis (*Perge et al., 2017*; *Sanchita et al., 2018*; *Zhao et al., 2018*). The biogenesis and function of such exogenous miRNAs are evidently health related (*Arnold et al., 2012*; *Izumi et al., 2012*; *Liu et al., 2012*; *Baier et al., 2014*).

## Discussion

In this study, bioinformatics analyses were employed to predict exogenous miRNAs that target human mRNAs. The miRNAs have been predicted as highly transportable candidates; several of them have identical sequences with their homologs in human (Table 2). It was found experimentally and *in silico* that hsa-miR-127-5p, hsa-miR-136, hsa-miR-431, hsa-miR-432, and hsa-miR-433 bind to mRNA of the human *RTL1* gene in identical positions with bta-miR-127, bta-miR-136, bta-miR-431, bta-miR-432, and bta-miR-433 (*Davis et al., 2005*; *Yurikova et al., 2019*).

Humans have a number of identical miRNAs for bta-miR-135a, bta-miR-136, bta-miR-432, bta-miR-127, bta-miR-431, bta-miR-433, bta-miR-1282, and bta-miR196a. The identical sequence may indicate a higher probability that the exogenous miRNA will regulate human genes after transportation into circulation. Moreover, miRNAs have been identified to be conserved across mammalian and non-mammalian species and this highlights that homologous miRNAs may share similar functional roles in common pathways in the evolutionary mechanism of distinct species (*Liu et al., 2012* *;* *van Herwijnen et al., 2018*). Human breast milk contains hsa-miR-136 and hsa-miR-135a, hsa-miR-432, hsa-miR-433 in colostrum, hsa-miR-196a and hsa-miR-431 in milk and colostrum (*Weber et al., 2010*). Previously 245 miRNAs have been found in cow milk by Chen et al. (*Chen et al., 2010*). The bta-miR-135a, bta-miR-136, bta-miR-127-3p, bta-miR-196a, and bta-miR-432 were found in bovine milk and colostrum (Table 2) (*Chen et al., 2010*). These experiments confirm that these five miRNAs are expressed in such mature milk-specific miRNAs and colostrum-specific miRNAs. Overall, 95% of the miRNA expressed in human milk was also expressed in cow milk. Milk-derived miRNAs can be transferred from bovine milk to humans and regulate gene expression in target tissues and cells. Milk derived miRNAs can enter normal and malignant cells and can regulate biological signals (*Golan-Gerstl et al., 2017;* *Zempleni et al., 2018*). Most milk miRNAs can affect human protein-coding genes. The bta-miR-151-5p, bta-miR-151-3p, bta-miRNA-320 have BSs in 11 genes, while bta-miRNA-345-5p, bta-miRNA-614, bta-miRNA-1296b and bta-miRNA-149 have BSs in 12, 14, 15 and 26 genes, respectively. The bta-miR-574-5p from cow’s milk had from one to 25 repeating BSs in mRNAs of 209 human genes, indicating this miRNA’s significant biological role. Clearly, experiments are needed next to determine possibly exosomal transport pathways. Therefore, future studies will need to address if milk-derived exosomal miRNAs can be transferred from bovine milk to humans and regulate gene expression in humans. Alternatively, such exosomes may act directly on cells such as MECs (mammary epithelial cells): Kelleher et al. could recently show that miRNAs are involved in MEC signaling and intriguingly, also appear to be required for healthy breast functions (*Kelleher et al., 2019*).

This study identified three miRNAs: bta-miR-11975, bta-miR-11976 and bta-miR-2885, in a cluster that targets multiple human-associated genes. The bta-miR-11976 and bta-miR-11975 recently discovered in a bovine (*Morenikeji et al., 2020*). The data obtained in our work on the possible effect of these miRNAs on human genes that cause many diseases provide a basis for further investigation of the effect of these exogenous miRNAs on human genes. Thus, from the data shown in Supplementary Table S11, it can be seen that almost half of the target genes are transcription factors, which makes it difficult to establish the effect of miRNAs on these target genes, since transcription factors can affect the expression of genes that control various functions. The miRNAs can affect oncogenes or onco-suppressors, showing the opposite effect on oncogenesis. For example, an increased level of miR-222 expression suppressed the effect of a breast cancer tumor suppressor, and a decreased miR-222 level increased the expression level of a tumor suppressor (*Said et al., 2021*). Increased expression of miRNA-200a-3p promotes the growth and development of gastric cancer tumors by suppressing the tumor suppressor (*Li et al., 2021*). Conversely, miR-345-5p plays the role of a tumor suppressor in lung adenocarcinoma cells, suppressing the oncogene (*Zhou Y. et al., 2021*).

Conducting *in silico* research leads to the question of how reliable the results are. The MirTarget program has been used in several studies and predicted the results of earlier and later studies. The high predictive advantage of the MirTarget program lies in the fact that it finds BSs for the entire nucleotide sequence of miRNA and determines important quantitative characteristics of the interaction of miRNA with mRNA. This approach in determining the properties of the interaction of exosomal miRNA with mRNA made it possible to reveal fundamentally new properties of miRNA forming in mRNA clusters to reduce the length of the 5′UTR and CDS regions (*Kondybayeva et al., 2018*; *Aisina et al., 2019*; *Mukushkina et al., 2020*; *Akimniyazova et al., 2021*; *Kamenova et al., 2021*). Studying the interaction of exogenous miRNAs with mRNA genes of humans *in silico* makes it possible to significantly reduce the material and time costs for determining effective associations of miRNAs and their target genes. A fundamentally important result of this work is to establish the possibility of the effect of exogenous miRNAs on the expression of human genes, including candidate genes for socially significant human diseases.

## Data Availability

The datasets presented in this study can be found in online repositories. The names of the repository/repositories and accession number(s) can be found in the article/Supplementary Material.
