# Supplementary material for: Identification of Bovine miRNAs with the Potential to Affect Human Gene Expression
Source: Front Genet. 2022 Jan 11;12:705350. doi: 10.3389/fgene.2021.705350 (PMC8787201; doi:10.3389/fgene.2021.705350)
Supplement: Supplementary file 1 [file Table15.DOCX]

**Supplementary Table S11** Functions of human target genes with bta-miR-11976, bta-miR-11975, bta-miR-2885.

| **ID** | **Gene** | **Function** | **PMID** |
| --- | --- | --- | --- |
| 84913 | *ATOH8* | Atoh8 is a bHLH transcription factor expressed in pancreas, skeletal muscle, the nervous system, and cardiovascular tissues during embryological development. Although it has been implicated in the regulation of pancreatic and endothelial cell differentiation, the phenotypic consequences of Atoh8 loss are uncertain. | 28036274  Breast cancer |
| 170302 | *ARX* | This gene is a homeobox-containing gene expressed during development. The expressed protein contains two conserved domains, a C-peptide (or aristaless domain) and the prd-like class homeobox domain. It is a member of the group-II aristaless-related protein family whose members are expressed primarily in the central and/or peripheral nervous system. This gene is thought to be involved in CNS development. Expansion of a polyalanine tract and other mutations in this gene cause X-linked cognitive disability and epilepsy. | 28627419  Interneuron development |
| 54897 | *CASZ1* | this gene is a zinc finger transcription factor. The encoded protein may function as a tumor suppressor, and single nucleotide polymorphisms in this gene are associated with blood pressure variation. | 27693370  Cardiac development  heart disease |
| 56936 | *CCDC177* | Coiled-coil domain containing 177 | 26646589  Neiroblastoma |
| 1050 | *CEBPA* | This intronless gene encodes a transcription factor that contains a basic leucine zipper (bZIP) domain and recognizes the CCAAT motif in the promoters of target genes. The encoded protein functions in homodimers and also heterodimers with CCAAT/enhancer-binding proteins beta and gamma. Activity of this protein can modulate the expression of genes involved in cell cycle regulation as well as in body weight homeostasis. Mutation of this gene is associated with acute myeloid leukemia. | 28637622  Myeloid leukemia. |
| 1501 | *CTNND2* | Delta-catenin (CTNND2), which have been mapped to the "critical regions", are potentially involved in cerebral development and their deletion may be associated with mental retardation in CdCS patients. | 32352179  Lyposarcoma |
| 1107 | *CHD3* | This gene encodes a member of the CHD family of proteins which are characterized by the presence of chromo (chromatin organization modifier) domains and SNF2-related helicase/ATPase domains. This protein is one of the components of a histone deacetylase complex referred to as the Mi-2/NuRD complex which participates in the remodeling of chromatin by deacetylating histones. Chromatin remodeling is essential for many processes including transcription. Autoantibodies against this protein are found in a subset of patients with dermatomyositis. | 30397230  Neurodevelopmental syndrome |
| 1750 | *DLX6* | This gene encodes a member of a homeobox transcription factor gene family similiar to the Drosophila distal-less gene. This family is comprised of at least 6 different members that encode proteins with roles in forebrain and craniofacial development. | 31612030  Lung cancer |
| 63950 | *DMRTA2* | DMRTA2 encodes doublesex and mab-3-related transcription factor a2, a transcription factor key to the development of the dorsal telencephalon. | 26757254 Corticaldevelopment |
| 9162 | *DGKI* | This gene is a member of the type IV diacylglycerol kinase subfamily. Diacylglycerol kinases regulate the intracellular concentration of diacylglycerol through its phosphorylation, producing phosphatidic acid. The specific role of the enzyme encoded by this gene is undetermined, however, it may play a crucial role in the production of phosphatidic acid in the retina or in recessive forms of retinal degeneration. | 32733904  Gastriccancer |
| 2304 | *FOXE1* | This intronless gene encodes a protein that belongs to the forkhead family of transcription factors. Members of this family contain a conserved 100-amino acid DNA-binding 'forkhead' domain. The encoded protein functions as a thyroid transcription factor that plays a role in thyroid morphogenesis. Mutations in this gene are associated with the Bamforth-Lazarus syndrome, and with susceptibility to nonmedullary thyroid cancer-4. | 25950909  ThyroidCancer |
| 2295 | *FOXF2* | FOXF2 encodes forkhead box F2, one of many human homologues of the Drosophila melanogaster transcription factor forkhead. FOXF2 is expressed in lung and placenta, and has been shown to transcriptionally activate several lung-specific genes. | 25848863  Breastcancer |
| 752 | *FMNL1* | Formin-like 1 (FMNL1) is a member of Formin family proteins which are the actin nucleators. Although FMNL1 activities have been shown to be essential for cell adhesion, cytokinesis, cell polarization and migration in mitosis, the functional roles of mammalian FMNL1 during oocyte meiosis remain uncertain. In this study, we investigated the functions of FMNL1 in mouse oocytes using specific morpholino (MO) microinjection and live cell imaging. | 33324547  Cell carcinoma |
| 64839 | *FBXL17* | Members of the F-box protein family, such as FBXL17, are characterized by an approximately 40-amino acid F-box motif. SCF complexes, formed by SKP1 (MIM 601434), cullin (see CUL1; MIM 603134), and F-box proteins, act as protein-ubiquitin ligases. F-box proteins interact with SKP1 through the F box, and they interact with ubiquitination targets through other protein interaction domains | 27234298  Medulloblastoma |
| 2297 | *FOXD1* | This gene belongs to the forkhead family of transcription factors which are characterized by a distinct forkhead domain. Studies of the orthologous mouse protein indicate that it functions in kidney development by promoting nephron progenitor differentiation, and it also functions in the development of the retina and optic chiasm. It may also regulate inflammatory reactions and prevent autoimmunity. | 33403027  Cellcarcinoma |
| 2290 | *FOXG1* | This locus encodes a member of the fork-head transcription factor family. The encoded protein, which functions as a transcriptional repressor, is highly expressed in neural tissues during brain development. Mutations at this locus have been associated with Rett syndrome and a diverse spectrum of neurodevelopmental disorders defined as part of the FOXG1 syndrome. This gene is disregulated in many types of cancer and is the target of multiple microRNAs that regulate the proliferation of tumor cells. | 31824897  Neurodevelop-mentaldisorders |
| 221937 | *FOXK1* | FOXK1 was highly expressed in HC and cell lines, which was associated with tumor invasion, regional lymph node metastasis, tumor recurrence and poor prognosis. Silencing FOXK1 in HC cells inhibited invasion and migration, upregulated E-cadherin, and downregulated vimentin, matrix metallopeptidase 9 and Twist in HC cells. | 33300075  Cholangiocar-cinoma |
| 9568 | *GABBR2* | The multi-pass membrane protein encoded by this gene belongs to the G-protein coupled receptor 3 family and GABA-B receptor subfamily. The GABA-B receptors inhibit neuronal activity through G protein-coupled second-messenger systems, which regulate the release of neurotransmitters, and the activity of ion channels and adenylyl cyclase. This receptor subunit forms an active heterodimeric complex with GABA-B receptor subunit 1, neither of which is effective on its own. Allelic variants of this gene have been associated with nicotine dependence. | 32807774  Autismspectrumdisorder |
| 2637 | *GBX2* | The transcription factor gastrulation-brain-homeobox 2 (Gbx2) in NC cell migration and positioning of motor neurons in the developing hindbrain. | 32466118  Cardiovascular |
| 285601 | *GPR150* | This gene encodes an orphan member of the class A rhodopsin-like family of G-protein-coupled receptors (GPCRs). Within the rhodopsin-like family, this gene is a member of the vasopressin-like subfamily that also includes vasopressin and oxytocin receptors. The silencing of this gene, due to promoter methylation, is associated with ovarian cancer progression. All GPCRs have a transmembrane domain that includes seven transmembrane alpha-helices. A general feature of GPCR signaling is the agonist-induced conformational change in the receptor, leading to activation of the heterotrimeric G protein. The activated G protein then binds to and activates numerous downstream effector proteins, which generate second messengers that mediate a broad range of cellular and physiological processes. | 25908269  Liverdisease |
| 54112 | *GPR88* | The protein encoded by this gene is a G protein-coupled receptor found almost exclusively in the striatum, a brain structure that controls motor function and cognition. Defects in this gene have been associated with chorea, speech delay, and learning difficulties, as well as some neuropsychiatric disorders. | 31666199  Parkinson'sdisease |
| 610 | *HCN2* | The protein encoded by this gene is a hyperpolarization-activated cation channel involved in the generation of native pacemaker activity in the heart and in the brain. The encoded protein is activated by cAMP and can produce a fast, large current. Defects in this gene were noted as a possible cause of some forms of epilepsy. | 29047147  Epilepsy |
| 3199 | *HOXA2* | In vertebrates, the genes encoding the class of transcription factors called homeobox genes are found in clusters named A, B, C, and D on four separate chromosomes. Expression of these proteins is spatially and temporally regulated during embryonic development. This gene is part of the A cluster on chromosome 7 and encodes a DNA-binding transcription factor which may regulate gene expression, morphogenesis, and differentiation. The encoded protein may be involved in the placement of hindbrain segments in the proper location along the anterior-posterior axis during development. | 30033361  Prostatecancer |
| 3209 | *HOXA13* | In vertebrates, the genes encoding the class of transcription factors called homeobox genes are found in clusters named A, B, C, and D on four separate chromosomes. Expression of these proteins is spatially and temporally regulated during embryonic development. This gene is part of the A cluster on chromosome 7 and encodes a DNA-binding transcription factor which may regulate gene expression, morphogenesis, and differentiation. Expansion of a polyalanine tract in the encoded protein can cause hand-foot-uterus syndrome, also known as hand-foot-genital syndrome. | 28766961  Prostate cancer |
| 3064 | *HTT* | The huntingtin gene is widely expressed and is required for normal development. It is expressed as 2 alternatively polyadenylated forms displaying different relative abundance in various fetal and adult tissues. The larger transcript is approximately 13.7 kb and is expressed predominantly in adult and fetal brain whereas the smaller transcript of approximately 10.3 kb is more widely expressed. The genetic defect leading to Huntington's disease may not necessarily eliminate transcription, but may confer a new property on the mRNA or alter the function of the protein. One candidate is the huntingtin-associated protein-1, highly expressed in brain, which has increased affinity for huntingtin protein with expanded polyglutamine repeats. This gene contains an upstream open reading frame in the 5' UTR that inhibits expression of the huntingtin gene product through translational repression | 26938440  Huntington'sdisease |
| 64207 | *IRF2BPL* | Interferon regulatory factor 2 binding protein-like (IRF2BPL) encodes a member of the IRF2BP family of transcriptional regulators. Currently the biological function of this gene is obscure, and the gene has not been associated with a Mendelian disease. | 30193138  Neyrological phenotypes |
| 8660 | *IRS2* | This gene encodes the insulin receptor substrate 2, a cytoplasmic signaling molecule that mediates effects of insulin, insulin-like growth factor 1, and other cytokines by acting as a molecular adaptor between diverse receptor tyrosine kinases and downstream effectors. The product of this gene is phosphorylated by the insulin receptor tyrosine kinase upon receptor stimulation, as well as by an interleukin 4 receptor-associated kinase in response to IL4 treatment. | 30250623  Cholangiocarcinoma |
| 153572 | *IRX2* | IRX2 is a member of the Iroquois homeobox gene family. Members of this family appear to play multiple roles during pattern formation of vertebrate embryos. | 26560478  Breast cancer |
| 79191 | *IRX3* | IRX3 is a member of the Iroquois homeobox gene family (see IRX1; MIM 606197) and plays a role in an early step of neural development (Bellefroid et al., 1998 [PubMed 9427753]). Members of this family appear to play multiple roles during pattern formation of vertebrate embryos | 27222047  hepatocellularcarcinoma |
| 50805 | *IRX4* | IRX4 was the first identified cardiac transcription factor that is restricted to the ventricles at all stages of heart development. Irx4-deficient mice show ventricular dysfunction and develop cardiomyopathy. | 21544582  Heartdisease |
| 10265 | *IRX5* | This gene encodes a member of the iroquoishomeobox gene family, which are involved in several embryonic developmental processes. Knockout mice lacking this gene show that it is required for retinal cone bipolar cell differentiation, and that it negatively regulates potassium channel gene expression in the heart to ensure coordinated cardiac repolarization. Alternatively spliced transcript variants encoding different isoforms have been found for this gene. | 32898233  Cardiacdiseases |
| 3727 | *JUND* | The protein encoded by this intronless gene is a member of the JUN family, and a functional component of the AP1 transcription factor complex. This protein has been proposed to protect cells from p53-dependent senescence and apoptosis. Alternative translation initiation site usage results in the production of different isoforms | 26391109  Cardiovasculardisease |
| 254251 | *LCORL* | This gene encodes a transcription factor that appears to function in spermatogenesis. Polymorphisms in this gene are associated with measures of skeletal frame size and adult height. Alternative splicing results in multiple transcript variants. | 29860282  Alzheimer'sdisease |
| 375612 | *LHFPL3* | This gene is a member of the lipoma HMGIC fusion partner (LHFP) gene family, which is a subset of the superfamily of tetraspan transmembrane protein encoding genes. Mutations in one LHFP-like gene result in deafness in humans and mice, and a second LHFP-like gene is fused to a high-mobility group gene in a translocation-associated lipoma. A partial gene fragment named LHFPL4 corresponds to a portion of the first exon of this gene. | 32753471  Melanoma |
| 4016 | *LOXL1* | The prototypic member of the family is essential to the biogenesis of connective tissue, encoding an extracellular copper-dependent amine oxidase that catalyzes the first step in the formation of crosslinks in collagen and elastin. The encoded preproprotein is proteolytically processed to generate the mature enzyme. A highly conserved amino acid sequence at the C-terminus end appears to be sufficient for amine oxidase activity, suggesting that each family member may retain this function. The N-terminus is poorly conserved and may impart additional roles in developmental regulation, senescence, tumor suppression, cell growth control, and chemotaxis to each member of the family. | 31732974  Pancreaticcancer |
| 4052 | *LTBP1* | The secretion and activation of TGF-betas is regulated by their association with latency-associated proteins and with latent TGF-beta binding proteins. The product of this gene targets latent complexes of transforming growth factor beta to the extracellular matrix, where the latent cytokine is subsequently activated by several different mechanisms. | 33246463  Glioblastoma multiforme |
| 4204 | *MECP2* | MECP2 is dispensible in stem cells, but is essential for embryonic development. MECP2 gene mutations are the cause of most cases of Rett syndrome, a progressive neurologic developmental disorder and one of the most common causes of cognitive disability in females. | 32698189  Neurodevelopmentaldisorder |
| 10893 | *MMP24* | This gene encodes a member of the peptidase M10 family of matrix metalloproteinases (MMPs). Proteins in this family are involved in the breakdown of extracellular matrix in normal physiological processes, such as embryonic development, reproduction, and tissue remodeling, as well as in disease processes, such as arthritis and metastasis. | 26322584  Alzheimerdisease |
| 3110 | *MNX1* | This gene encodes a nuclear protein, which contains a homeobox domain and is a transcription factor. Mutations in this gene result in Currarino syndrome, an autosomic dominant congenital malformation. Alternatively spliced transcript variants encoding different isoforms have been found for this gene. | 30614606  Colorectalcancer |
| 340719 | *NANOS1* | This gene encodes a CCHC-type zinc finger protein that is a member of the family. This protein co-localizes with the RNA-binding protein pumilio RNA-binding family member 2 and may be involved in regulating translation as a post-transcriptional repressor. Mutations in this gene are associated with spermatogenic impairment. | 31694904  Breastcancer |
| 159296 | *NKX2-3* | This gene encodes a homeodomain-containing transcription factor. The encoded protein is a member of the NKX family of homeodomain transcription factors. Studies of similar proteins in mouse and rat have indicated a potential role in cellular differentiation | 26957275  Heart desease |
| 5455 | *POU3F3* | This gene encodes a POU-domain containing protein that functions as a transcription factor. The encoded protein recognizes an octamer sequence in the DNA of target genes. This protein may play a role in development of the nervous system. | 32236821  Parkinson's disease |
| 652991 | *SKOR2* | The Ski/Sno family transcriptional co-repressor 2 (Skor2) gene | 19934318 Cellcarcinoma |
| 57419 | *SLC24A3* | Plasma membrane sodium/calcium exchangers are an important component of intracellular calcium homeostasis and electrical conduction. Potassium-dependent sodium/calcium exchangers such as SLC24A3 are believed to transport 1 intracellular calcium and 1 potassium ion in exchange for 4 extracellular sodium ions | 31083655  Meningiomas |
| 55084 | *SOBP* | The protein encoded by this gene is a nuclear zinc finger protein that is involved in development of the cochlea. Defects in this gene have also been linked to intellectual disability. | 29304828   Lungcancer |
| 6666 | *SOX12* | SOX transcription factors have diverse tissue-specific expression patterns during early development and have been proposed to act as target-specific transcription factors and/or as chromatin structure regulatory elements. The protein encoded by this gene was identified as a SOX family member based on conserved domains, and its expression in various tissues suggests a role in both differentiation and maintenance of several cell types. | 30858360  Colorectalcancer |
| 11166 | *SOX21* | SRY-related HMG-box (SOX) genes encode a family of DNA-binding proteins containing a 79-amino acid HMG (high mobility group) domain that shares at least 50% sequence identity with the DNA-binding HMG box of the SRY protein (MIM 480000). | 30912129  Cervicalcancer |
| 221833 | *SP8* | The protein encoded by this gene is an SP family transcription factor that in mouse has been shown to be essential for proper limb development. | 32824198  Hepatoblastomaliver tumor |
| 100507588 | *TGFBR3L* | Transforming growth factor beta-receptor 3 like*TGFBR3L* showed membranous immunolabeling and was found to be gonadotroph cell lineage-specific, verified by co-expression with SF1 and FSH/LH staining in both tumour and non-neoplastic anterior pituitary tissues. | 33396509  Neuroendocrine tumours |
| 64061 | *TSPYL2* | This gene encodes a member of the testis-specific protein Y-encoded, TSPY-like/SET/nucleosome assembly protein-1 superfamily. The encoded protein is localized to the nucleolus where it functions in chromatin remodeling and as an inhibitor of cell-cycle progression. | 26059843  Neurodevelopmentaldisorders |
| 440730 | *TRIM67* | Tripartite motif (TRIM) family proteins participate in a variety of important cellular processes, including apoptosis, cell-cycle arrest, DNA repair, and senescence. | 31239268  Colorectal cancer |
| 286262 | *TPRN* | Tprn encodes the taperin protein, which is concentrated in the tapered region of hair cell stereocilia in the inner ear. In humans, TPRN mutations cause autosomal recessive nonsyndromic deafness (DFNB79) by an unknown mechanism. | 30277474  Myeloma |
| 340260 | *UNCX* | This gene encodes a homeobox transcription factor that is involved in somitogenesis and neurogenesis and is required for the maintenance and differentiation of specific elements of the axial skeleton. | 28855354  Non-small cell lung cancer |
| 54877 | *ZCCHC2* | The zinc-finger protein ZCCHC2 as a critical negative regulator of c-Myc-associated tumorigenesis. Knockout of ZCCHC2 promoted retinoblastoma cell proliferation, whereas ZCCHC2 overexpression had the opposite effect. | 31677785 Retinoblastoma tumorigenesis |
| 7547 | *ZIC3* | This gene encodes a member of the ZIC family of C2H2-type zinc finger proteins. This nuclear protein probably functions as a transcription factor in early stages of left-right body axis formation. Mutations in this gene cause X-linked visceral heterotaxy, which includes congenital heart disease and left-right axis defects in organs. | 21858219 Heartdisease |
| 85416 | *ZIC5* | This gene encodes a member of the ZIC family of C2H2-type zinc finger proteins. The encoded protein may act as a transcriptional repressor.Elevated expression of this gene has been observed in various human cancers and may contribute to cancer progression. | 29628984  BreastCancer |
| 57688 | *ZSWIM6* | The zinc-finger SWIM domain-containing protein 6 (ZSWIM6) is a protein of unknown function that has been associated with schizophrenia and limited educational attainment by three independent genome-wide association studies. | 28433741 Neurodevelopmentaldisorders. |
| 195828 | *ZNF367* | ZNF367 was a transcription factor. ZNF367 is a member of the ZNF family, which is found to be overexpressed in adrenocortical carcinoma, malignant pheochromocytoma/paraganglioma, and thyroid cancer. | 32549756  Breast cancer |
| 80139 | *ZNF703* | Zinc finger protein 703 (ZNF703), a member of the NET family of transcription factors, has recently emerged as an important player in the development of several types of cancers. | 30361900  Thyroid Carcinoma |
| 55778 | *ZNF839* | Zinc finger protein 839 | 28138309  Colorectal Cancer |
