# Supplementary material for: Identification of Bovine miRNAs with the Potential to Affect Human Gene Expression
Source: Front Genet. 2022 Jan 11;12:705350. doi: 10.3389/fgene.2021.705350 (PMC8787201; doi:10.3389/fgene.2021.705350)
Supplement: Supplementary file 2 [file Table8.DOCX]

**Supplementary Table S4** Characteristics of interactions of bta-miRNA BSs in СDS mRNA of human genes in clusters with length of 21 nt

| **Gene** | **bta-miRNA** | **Start of**  **site, nt** | **ΔG,**  **kJ/mole** | **∆G/∆Gm_,_**  **%** | **Length,**  **nt** |
| --- | --- | --- | --- | --- | --- |
| *ARX* | bta-miR-11975 | 1505÷1511 (3) | -117÷-121 | 92÷95 | 20 |
|  | bta-miR-11976 | 1507, 1510 | -123 | 92 | 21 |
| *CEBPA* | bta-miR-11976 | 655 | -121 | 90 | 21 |
|  | bta-miR-11975 | 656 | -121 | 95 | 20 |
| *CHD3* | bta-miR-11975 | 221÷227 (3) | -115÷-121 | 90÷95 | 20 |
|  | bta-miR-11976 | 223, 226 | -123÷-127 | 92÷95 | 21 |
| *CTNND2* | bta-miR-11975 | 796÷805 (4) | -114÷-121 | 90÷95 | 20 |
|  | bta-miR-11976 | 799÷804 (3) | -121÷-127 | 90÷95 | 21 |
|  | bta-miR-2885 | 801, 804 | -110 | 93 | 19 |
|  | bta-miR-11976 | 871 | -121 | 90 | 21 |
|  | bta-miR-11975 | 872 | -114 | 90 | 20 |
| *DGKI* | bta-miR-11976 | 64, 73 | -121÷-123 | 90÷92 | 21 |
|  | bta-miR-11975 | 65÷74 (3) | -115÷-121 | 90÷95 | 20 |
| *GSG1L* | bta-miR-11976 | 277÷292 (6) | -121÷-127 | 90÷95 | 21 |
|  | bta-miR-11975 | 278÷293 (6) | -121 | 92÷95 | 20 |
| *HCN2* | bta-miR-11976 | 109, 115 | -121 | 90 | 21 |
|  | bta-miR-11975 | 116 | -121 | 95 | 20 |
|  | bta-miR-11975 | 152, 155 | -115÷-121 | 90÷95 | 20 |
|  | bta-miR-11976 | 154 | -121 | 90 | 21 |
|  | bta-miR-11975 | 2195, 2198 | -115 | 90 | 20 |
| *HTT* | bta-miR-11975 | 259÷268 (4) | -117÷-121 | 92 | 20 |
|  | bta-miR-11976 | 261÷267 (3) | -123÷-127 | 92÷95 | 21 |
|  | bta-miR-2885 | 261, 267 | -110 | 93 | 19 |
| *JUND* | bta-miR-11975 | 610÷616 (3) | -117 | 92÷95 | 20 |
|  | bta-miR-11976 | 612, 615 | -123 | 92 | 21 |
|  | bta-miR-2885 | 618 | -110 | 92 | 19 |
| *SKOR2* | bta-miR-11976 | 851÷860 (3) | -123÷-127 | 92÷95 | 21 |
|  | bta-miR-11975 | 852÷861 (3) | -117÷-121 | 90÷95 | 20 |
|  | bta-miR-11976 | 2078÷2093 (6) | -121÷-127 | 90÷95 | 21 |
|  | bta-miR-11975 | 2079÷2094 (6) | -115÷-121 | 90÷95 | 20 |
|  | bta-miR-2885 | 2080 | -121 | 95 | 19 |
| *SLC24A3* | bta-miR-11976 | 222÷231 (3) | -115÷-121 | 90÷95 | 21 |
|  | bta-miR-11975 | 223÷232 (3) | -115÷-117 | 90÷95 | 20 |
|  | bta-miR-2885 | 231, 234 | -110 | 93 | 19 |
| *SOBP* | bta-miR-11975 | 2733, 2736 | -115÷-121 | 90÷95 | 20 |
|  | bta-miR-11976 | 2735 | -121 | 90 | 21 |
| *TGFBR3L* | bta-miR-11976 | 681 | -125 | 94 | 21 |
|  | bta-miR-11975 | 682÷691 (3) | -115÷-125 | 90÷95 | 20 |
|  | bta-miR-2885 | 681, 690 | -108÷-110 | 91÷93 | 19 |
