# Supplementary material for: Identification of Bovine miRNAs with the Potential to Affect Human Gene Expression
Source: Front Genet. 2022 Jan 11;12:705350. doi: 10.3389/fgene.2021.705350 (PMC8787201; doi:10.3389/fgene.2021.705350)
Supplement: Supplementary file 3 [file Table12.DOCX]

**Supplementary Table S8** Characteristics of interactions of bta-miRNA with human 5′UTR mRNA containing the BS clusters with length of 21 nt.

| **Gene** | **bta-miRNA** | **Start of**  **site, nt** | **ΔG,**  **kJ/mole** | **∆G/∆Gm_,_**  **%** | **Length,**  **nt** |
| --- | --- | --- | --- | --- | --- |
| *ABCD3* | bta-miR-11976 | 45, 48 | -121 | 90 | 21 |
|  | bta-miR-11975 | 46, 49 | -115÷-121 | 90÷95 | 20 |
|  | bta-mir-2885 | 48 | -110 | 93 | 19 |
| *ANKH* | bta-miR-11976 | 39, 42 | -123 | 92 | 21 |
|  | bta-miR-11975 | 40, 43 | -117 ÷-121 | 92÷95 | 20 |
| *ANKRD13D* | bta-miR-11975 | 22÷37 (5) | -117÷-121 | 92÷95 | 20 |
|  | bta-miR-11976 | 24÷36 (4) | -123 | 92 | 21 |
| *C4orf19* | bta-miR-11975 | 73÷82 (4) | -114÷121 | 90÷95 | 20 |
|  | bta-miR-11976 | 75÷81 (3) | -121÷-127 | 92÷95 | 21 |
|  | bta-mir-2885 | 78, 81 | -110 | 93 | 19 |
| *CA10* | bta-miR-11975 | 772÷778 (3) | -117÷-121 | 92÷95 | 20 |
|  | bta-miR-11976 | 774, 777 | -123 | 92 | 21 |
| *DISP2* | bta-miR-11975 | 13÷28 (6) | -115÷-117 | 90÷92 | 20 |
|  | bta-miR-11976 | 15÷27 (5) | -121÷-127 | 90÷95 | 21 |
|  | bta-miR-2885 | 18, 24 | -110 | 93 | 19 |
| *HS3ST4* | bta-miR-11975 | 352÷358 (3) | -114÷-121 | 90÷95 | 20 |
|  | bta-miR-11976 | 354, 357 | -121÷-123 | 90÷92 | 21 |
|  | bta-miR-2885 | 357 | -110 | 92 | 19 |
| *JARID2* | bta-miR-11975 | 68÷74 (3) | -117÷-121 | 92÷95 | 20 |
|  | bta-miR-11976 | 70, 73 | -123 | 92 | 21 |
| *RGP1* | bta-miR-11975 | 31÷37 (3) | -115÷-121 | 90 | 21 |
|  | bta-miR-11976 | 33, 36 | -121÷-127 | 90÷95 | 19 |
|  | bta-miR-2885 | 33, 36 | -110 | 93 | 19 |
| *UBE2R2* | bta-miR-2885 | 541÷553 (3) | -110 | 93 | 19 |
|  | bta-miR-11976 | 542÷553 (4) | -121÷127 | 90÷95 | 21 |
|  | bta-miR-11975 | 542÷554 (7) | -115÷-121 | 90÷95 | 20 |
| *USP25* | bta-miR-11976 | 181÷190 (4) | -121÷-127 | 90÷95 | 21 |
|  | bta-miR-11975 | 182÷191 (4) | -115÷-121 | 90÷95 | 20 |
|  | bta-miR-2885 | 187, 190 | -110 | 93 | 19 |
