# Supplementary material for: Identification of Bovine miRNAs with the Potential to Affect Human Gene Expression
Source: Front Genet. 2022 Jan 11;12:705350. doi: 10.3389/fgene.2021.705350 (PMC8787201; doi:10.3389/fgene.2021.705350)
Supplement: Supplementary file 4 [file Table7.DOCX]

**Supplementary Table S3** Characteristics of interactions of bta-miRNA BSs in СDS mRNA of human genesin clusters with length of 18 nt

| **Gene** | **miRNA** | **Start of**  **site, nt** | **ΔG,**  **kJ/mole** | **∆G/∆G_m,_**  **%** | **Length,**  **nt** |
| --- | --- | --- | --- | --- | --- |
| *ATOH8* | bta-miR-11976 | 772 | -121 | 90 | 21 |
|  | bta-miR-11975 | 773 | -115 | 90 | 20 |
| *CASZ1* | bta-miR-11975 | 5330, 5333 | -115 | 90 | 20 |
|  | bta-miR-11976 | 5332 | -121 | 90 | 21 |
|  | bta-miR-2885 | 5332 | -110 | 93 | 19 |
| *GABBR2* | bta-miR-11976 | 276, 279 | -123 | 92 | 21 |
|  | bta-miR-11975 | 277÷286 (3) | -114÷-116 | 90÷92 | 20 |
|  | bta-miR-11975 | 487÷499 (4) | -117÷-121 | 92÷95 | 20 |
|  | bta-miR-11976 | 495, 498 | -123 | 92 | 21 |
| *GPR150* | bta-miR-11976 | 566, 569 | -123 | 92 | 21 |
|  | bta-miR-11975 | 567, 570 | -117 | 91 | 20 |
| *GPR88* | bta-miR-11975 | 1123 | -115 | 90 | 20 |
| *FAM117B* | bta-miR-11976 | 420 | -121 | 91 | 21 |
|  | bta-miR-11975 | 421, 427 | -115÷-117 | 90÷92 | 20 |
| *FBXL17* | bta-miR-11975 | 771÷783 (4) | -117 | 92 | 20 |
|  | bta-miR-11976 | 779, 782 | -123 | 92 | 21 |
| *FOXD1* | bta-miR-11976 | 1052 | -121 | 90 | 21 |
|  | bta-miR-11975 | 1053 | -115 | 90 | 20 |
| *FOXG1* | bta-miR-11975 | 424, 427 | -114 | 90 | 20 |
| *FOXK1* | bta-miR-11976 | 156 | -127 | 95 | 21 |
|  | bta-miR-11975 | 157÷166 (3) | -117÷-121 | 92÷95 | 20 |
|  | bta-miR-2885 | 156 | -110 | 93 | 19 |
| *HOXA2* | bta-miR-11975 | 609, 612 | -117 | 92 | 20 |
|  | bta-miR-11976 | 611 | -123 | 92 | 21 |
| *HOXA13* | bta-miR-11975 | 399÷408 (3) | -115 | 90 | 20 |
|  | bta-miR-11976 | 620 | -129 | 97 | 21 |
|  | bta-miR-11975 | 621 | -123 | 96 | 20 |
| *IRX2* | bta-miR-11975 | 450÷456 (3) | -115 | 90 | 20 |
|  | bta-miR-11976 | 452, 455 | -121 | 90 | 21 |
| *IRX3* | bta-miR-11976 | 1571, 1745 | -123, -121 | 92, 90 | 21 |
|  | bta-miR-11975 | 1572, 1578 | -117 | 92 | 20 |
| *IRX4* | bta-miR-11975 | 1242÷1248 (3) | -121 | 92÷95 | 20 |
|  | bta-miR-11976 | 1244, 1247 | -123 | 92 | 21 |
| *IRX5* | bta-miR-11976 | 207÷213(3) | -121 | 90 | 21 |
|  | bta-miR-11975 | 214 | -115 | 90 | 20 |
| *LCORL* | bta-miR-11975 | 146÷155 (3) | -115÷-117 | 90÷92 | 20 |
| *LHFPL3* | bta-miR-11975 | 134÷146 (5) | -115÷-117 | 90÷92 | 20 |
|  | bta-miR-11976 | 136÷142 (3) | -123 | 92 | 21 |
| *LOXL1* | bta-miR-11975 | 1116 | -115 | 90 | 20 |
| *LTBP1* | bta-miR-11975 | 320 | -117 | 92 | 20 |
| *MECP2* | bta-miR-11975 | 70 | -115 | 90 | 20 |
| *NANOS1* | bta-miR-11976 | 581, 587 | -123÷-127 | 92÷95 | 21 |
|  | bta-miR-11975 | 582÷588 (3) | -114÷-117 | 90÷92 | 20 |
|  | bta-miR-2885 | 581 | -110 | 92 | 19 |
| *POU3F3* | bta-miR-11975 | 304÷325 (8) | -115÷-117 | 90 | 20 |
|  | bta-miR-11976 | 309÷324 (6) | -123 | 92 | 21 |
|  | bta-miR-2885 | 309, 318 | -110÷-112 | 93÷94 | 19 |
|  | bta-miR-11975 | 583÷592 (4) | -117÷-121 | 92 | 20 |
|  | bta-miR-11976 | 585÷591 (3) | -121 | 90 | 21 |
|  | bta-miR-2885 | 591 | -110 | 93 | 19 |
| *SOX12* | bta-miR-11975 | 961, 964 | -115 | 90 | 20 |
| *SOX21* | bta-miR-11975 | 552÷564 (5) | -115÷-117 | 90÷92 | 20 |
|  | bta-miR-11976 | 554÷560 (3) | -123 | 92 | 21 |
| *SP8* | bta-miR-11976 | 553, 556 | -121÷-123 | 90 | 21 |
|  | bta-miR-11975 | 554÷569 (6) | -115÷-117 | 92 | 20 |
|  | bta-miR-11976 | 562÷568 (3) | -121 | 90 | 21 |
| *TMEM121* | bta-miR-11975 | 1030 | -117 | 91 | 20 |
| *TSPYL2* | bta-miR-11976 | 213÷219 (3) | -121÷-127 | 90÷95 | 21 |
|  | bta-miR-11975 | 214÷220 (3) | -114÷-121 | 90÷95 | 20 |
|  | bta-miR-2885 | 213, 219 | -110 | 93 | 19 |
| *TPRN* | bta-miR-11976 | 468 | -115 | 90 | 20 |
|  | bta-miR-11975 | 469, 471 | -121 | 90 | 21 |
| *UNCX* | bta-miR-11976 | 1041, 1050 | -121 | 90 | 21 |
|  | bta-miR-2885 | 1050 | -110 | 92 | 19 |
| *ZCCHC2* | bta-miR-11976 | 126, 128 | -121 | 90 | 21 |
|  | bta-miR-11975 | 126, 129 | -115 | 90 | 20 |
| *ZNF367* | bta-miR-11976 | 700 | -123 | 92 | 21 |
|  | bta-miR-11975 | 701 | -117 | 92 | 20 |
| *ZNF839* | bta-miR-11976 | 167 | -121 | 90 | 21 |
|  | bta-miR-11975 | 168, 171 | -115 | 90 | 20 |
