# Supplementary material for: Identification of Bovine miRNAs with the Potential to Affect Human Gene Expression
Source: Front Genet. 2022 Jan 11;12:705350. doi: 10.3389/fgene.2021.705350 (PMC8787201; doi:10.3389/fgene.2021.705350)
Supplement: Supplementary file 5 [file Table9.DOCX]

**Supplementary Table S5** Characteristics of interactions of bta-miRNA with human CDS mRNA containing the BS clusters with length of 24 nt

| **Gene** | **bta-miRNA** | **Start of**  **site, nt** | **ΔG,**  **kJ/mole** | **∆G/∆Gm_,_**  **%** | **Length,**  **nt** |
| --- | --- | --- | --- | --- | --- |
| *CCDC177* | bta-miR-11976 | 874÷889 (6) | -121÷-127 | 90÷95 | 21 |
|  | bta-miR-11975 | 875÷890 (6) | -115÷-121 | 90÷95 | 20 |
| *FMNL1* | bta-miR-11976 | 2002÷2014 (5) | -114÷-127 | 90÷95 | 21 |
|  | bta-miR-11975 | 2003÷2015 (5) | -114÷-121 | 90÷95 | 20 |
|  | bta-miR-2885 | 2008, 2011 | -110 | 93 | 19 |
| *GBX2* | bta-miR-11975 | 200÷206 (3) | -117 | 92 | 20 |
|  | bta-miR-11976 | 202, 205 | -123 | 92 | 21 |
| *IRS2* | bta-miR-11976 | 2593, 2596 | -121÷-127 | 90÷95 | 21 |
|  | bta-miR-11975 | 2594, 2597 | -121 | 95 | 20 |
|  | bta-miR-2885 | 2596, 2599 | -110 | 93 | 19 |
| *MEGF9* | bta-miR-11975 | 199÷208 (4) | -117÷-121 | 95 | 20 |
|  | bta-miR-11976 | 201÷207 (3) | -127 | 92÷95 | 21 |
|  | bta-miR-2885 | 201, 204 | -110 | 93 | 19 |
| *MMP24* | bta-miR-11975 | 39, 42 | -121 | 95 | 20 |
|  | bta-miR-2885 | 41, 44 | -110 | 93 | 19 |
| *SKIDA1* | bta-miR-11975 | 2917÷2932 (5) | -115÷-121 | 90 | 20 |
|  | bta-miR-11976 | 2922÷2931 (4) | -121-÷127 | 90÷95 | 21 |
|  | bta-miR-2885 | 2928÷2943 (4) | -108÷-110 | 91÷93 | 19 |
|  | bta-miR-11976 | 2946÷2964 (5) | -121÷-127 | 90÷95 | 21 |
|  | bta-miR-11975 | 2947÷2965 (6) | -114÷-121 | 90÷92 | 20 |
| *TRIM67* | bta-miR-11975 | 795÷801 (3) | -115 | 90 | 20 |
|  | bta-miR-11976 | 797, 800 | -121 | 90÷95 | 21 |
| *ZIC3* | bta-miR-11975 | 635÷650 (5) | -116÷121 | 91÷95 | 20 |
|  | bta-miR-11976 | 640÷649 (4) | -123÷-127 | 95 | 21 |
|  | bta-miR-2885 | 643, 646 | -110 | 93 | 19 |
