# Supplementary material for: Identification of Bovine miRNAs with the Potential to Affect Human Gene Expression
Source: Front Genet. 2022 Jan 11;12:705350. doi: 10.3389/fgene.2021.705350 (PMC8787201; doi:10.3389/fgene.2021.705350)
Supplement: Supplementary file 6 [file Table5.DOCX]

**Supplementary Table S1** Characteristics of interactions of milk bta-miRNAs with

human mRNA genes

| bta-miRNA | Gene | Start of site, nt | Region of miRNA | ∆G,  kJ/mole | ∆G/∆G_m_, % | Length,  nt |
| --- | --- | --- | --- | --- | --- | --- |
| bta-let-7a-3p | *HTR7* | 2909 | 3'UTR | -96 | 92 | 21 |
|  | *USP46* | 7361 | 3'UTR | -98 | 94 | 21 |
| bta-let-7b | *GPR112* | 734 | CDS | -104 | 91 | 22 |
|  | *GPR85* | 1212 | CDS | -104 | 91 | 22 |
|  | *PFKFB1* | 783 | CDS | -104 | 91 | 22 |
| bta-let-7c | *DHX9* | 3517 | CDS | -102 | 91 | 22 |
| bta-let-7d | *HIF3A* | 2800 | 3'UTR | -104 | 92 | 22 |
|  | *CPA1* | 424 | CDS | -102 | 91 | 22 |
| bta-let-7e | *EPB41L4A* | 2300 | CDS | -100 | 92 | 21 |
| bta-let-7f | *CCNYL1* | 3594 | 3'UTR | -100 | 92 | 22 |
|  | *PFKFB1* | 783 | CDS | -98 | 90 | 22 |
| bta-let-7g | *TM2D1* | 517 | CDS | -102 | 92 | 22 |
| bta-let-7i | *FAM184A* | 3631 | 3'UTR | -102 | 91 | 22 |
| bta-miR-106a | *PLEKHM1* | 1215 | CDS | -102 | 91 | 22 |
| bta-miR-106b | *PLEKHM1* | 4123 | 3'UTR | -100 | 92 | 21 |
| bta-miR-107 | *IFRD2* | 1565 | CDS | -106 | 91 | 22 |
|  | *ANKUB1* | 1035 | CDS | -106 | 91 | 22 |
|  | *MGAT5* | 2381 | 3'UTR | -106 | 91 | 22 |
| bta-miR-122 | *KCNIP2* | 2371 | 3'UTR | -104 | 91 | 22 |
|  | *STARD9* | 3287 | CDS | -104 | 91 | 22 |
| bta-miR-1249 | *SART3* | 1602 | CDS | -110 | 90 | 22 |
|  | *C16orf89* | 144 | 5'UTR | -110 | 90 | 22 |
|  | *FAM163A* | 1642 | 3'UTR | -110 | 90 | 22 |
|  | *KAT2B* | 320 | 5'UTR | -110 | 90 | 22 |
|  | *KCNQ3* | 78 | 5'UTR | -110 | 90 | 22 |
| bta-miR-1277 | *WWP1* | 3298 | 3'UTR | -93 | 92 | 22 |
|  | *APOOL* | 1137 | 3'UTR | -91 | 90 | 22 |
| bta-miR-128 | *IQCG* | 825 | CDS | -102 | 92 | 21 |
| bta-miR-129-5p | *CCDC92* | 974 | CDS | -110 | 93 | 22 |
|  | *POLRMT* | 199 | CDS | -108 | 91 | 22 |
|  | *SMARCA2* | 1742 | CDS | -108 | 91 | 22 |
| bta-miR-1296 | *ARHGEF10L* | 1200 | CDS | -110 | 90 | 22 |
|  | *DDR2* | 667 | CDS | -115 | 93 | 22 |
|  | *HCLS1* | 338 | CDS | -110 | 90 | 22 |
|  | *HCN4* | 5078 | 3'UTR | -110 | 90 | 22 |
|  | *HOXB2* | 867 | CDS | -110 | 90 | 22 |
|  | *MICAL1* | 2295 | CDS | -110 | 90 | 22 |
|  | *MLL2* | 9261 | CDS | -110 | 90 | 22 |
|  | *SAFB* | 148 | 5'UTR | -110 | 90 | 22 |
|  | *TLCD2* | 12 | 5'UTR | -110 | 90 | 22 |
|  | *TMPRSS13* | 2161 | 3'UTR | -110 | 90 | 22 |
|  | *TOM1L2* | 14 | 5'UTR | -110 | 90 | 22 |
|  | *VSIG10L* | 2067 | CDS | -110 | 90 | 22 |
|  | *WDR73* | 707 | CDS | -110 | 90 | 22 |
|  | *WNT8A* | 746 | CDS | -110 | 90 | 22 |
|  | *ZC3H4* | 2153 | CDS | -110 | 90 | 22 |
| bta-miR-1306 | *KLF16* | 2766 | 3'UTR | -108 | 91 | 21 |
|  | *TPPP* | 5821 | 3'UTR | -110 | 93 | 21 |
|  | *VPS52* | 1819 | CDS | -108 | 91 | 21 |
| bta-miR-130a | *CLCN1* | 160 | CDS | -102 | 91 | 22 |
| bta-miR-130b | *FRMD7* | 2532 | 3'UTR | -104 | 91 | 22 |
|  | *SLC30A3* | 334 | CDS | -104 | 91 | 22 |
| bta-miR-132 | *PTCHD3* | 2426 | 3'UTR | -106 | 91 | 22 |
| bta-miR-133a | *C16orf58* | 1045 | CDS | -108 | 91 | 22 |
|  | *GPR179* | 426 | CDS | -108 | 91 | 22 |
|  | *TMEM71* | 607 | CDS | -113 | 95 | 22 |
| bta-miR-135a | *FHL5* | 2660 | 3'UTR | -104 | 92 | 23 |
|  | *GLYCTK* | 2812 | 3'UTR | -113 | 100 | 23 |
| bta-miR-136 | *CLDN15* | 37 | 5'UTR | -104 | 91 | 23 |
|  | *RTL1* | 110 | CDS | -115 | 100 | 23 |
| bta-miR-141 | *SSMEM1* | 554 | CDS | -102 | 91 | 22 |
|  | *ZZEF1* | 1308 | CDS | -102 | 91 | 22 |
| bta-miR-142-3p | *RRAGB* | 1701 | CDS | -100 | 90 | 22 |
|  | *TEX15* | 6569 | CDS | -102 | 92 | 22 |
|  | *UBXN10* | 2831 | 3'UTR | -102 | 92 | 22 |
| bta-miR-145 | *ARNTL* | 1867 | CDS | -110 | 90 | 23 |
|  | *COL4A4* | 3566 | CDS | -110 | 90 | 23 |
|  | *PRICKLE4* | 1024 | CDS | -113 | 91 | 23 |
|  | *SNX24* | 951 | 3'UTR | -110 | 90 | 23 |
|  | *WWOX* | 1154 | CDS | -110 | 90 | 23 |
| bta-miR-148a | *LAMA4* | 5896 | 3'UTR | -102 | 91 | 22 |
| bta-miR-148b | *LRRC34* | 1087 | CDS | -102 | 91 | 22 |
| bta-miR-148b | *GNB5* | 382 | CDS | -102 | 91 | 22 |
| bta-miR-149-3p | *ANKRD17* | 68 | 5'UTR | -119 | 92 | 22 |
|  | *C17orf104* | 28 | 5'UTR | -119 | 92 | 22 |
|  | *CASC3* | 41 | 5'UTR | -117 | 90 | 22 |
|  | *CASR* | 3300 | CDS | -117 | 90 | 22 |
|  | *CCDC105* | 1662 | 3'UTR | -117 | 90 | 22 |
|  | *CHRD* | 141 | 5'UTR | -117 | 90 | 22 |
|  | *CPLX1* | 619 | 3'UTR | -117 | 90 | 22 |
|  | *DGAT1* | 3161 | 3'UTR | -117 | 90 | 22 |
|  | *HAMP* | 336 | 3'UTR | -117 | 90 | 22 |
|  | *HNRNPA0* | 87 | 5'UTR | -117 | 90 | 22 |
|  | *KIAA0355* | 3088 | CDS | -117 | 90 | 22 |
|  | *MAP3K10* | 266 | 5'UTR | -117 | 90 | 22 |
|  | *MAPK8IP3* | 5332 | 3'UTR | -117 | 90 | 22 |
|  | *MARK2* | 3906 | 3'UTR | -117 | 90 | 22 |
|  | *MLLT10* | 91 | 5'UTR | -121 | 93 | 22 |
|  | *MUC2* | 8476 | 3'UTR | -117 | 90 | 22 |
|  | *MUC5B* | 4375 | CDS | -117 | 90 | 22 |
|  | *NCOR2* | 8232 | 3'UTR | -119 | 92 | 22 |
|  | *NFAM1* | 2273 | 3'UTR | -119 | 92 | 22 |
|  | *NKD2* | 1725 | 3'UTR | -117 | 90 | 22 |
|  | *PHOX2A* | 143 | 5'UTR | -117 | 90 | 22 |
|  | *PHTF1* | 184 | 5'UTR | -117 | 90 | 22 |
|  | *RENBP* | 1399 | CDS | -117 | 90 | 22 |
|  | *SERGEF* | 31 | 5'UTR | -117 | 90 | 22 |
|  | *TIMP2* | 210 | 5'UTR | -117 | 90 | 22 |
|  | *VAT1* | 4 | 5'UTR | -117 | 90 | 22 |
| bta-miR-150 | *TRPC1* | 1459 | CDS | -110 | 90 | 23 |
| bta-miR-150 | *SLC35C1* | 1722 | CDS | -110 | 90 | 23 |
| bta-miR-151-3p | *ARRDC2* | 1676 | 3'UTR | -104 | 92 | 21 |
|  | *CACNG8* | 3755 | 3'UTR | -102 | 91 | 21 |
|  | *CECR2* | 3410 | CDS | -104 | 92 | 21 |
|  | *DIS3* | 4266 | 3'UTR | -102 | 91 | 21 |
|  | *GKAP1* | 71 | 5'UTR | -106 | 94 | 21 |
|  | *KCNK6* | 1619 | 3'UTR | -102 | 91 | 21 |
|  | *MCF2L* | 995 | CDS | -102 | 91 | 21 |
|  | *SEPT8* | 2767 | 3'UTR | -110 | 98 | 21 |
|  | *TRIM37* | 2834 | CDS | -102 | 91 | 21 |
|  | *TTC40* | 220 | CDS | -102 | 91 | 21 |
|  | *UCK1* | 1721 | 3'UTR | -102 | 91 | 21 |
| bta-miR-151-5p | *DCAF10* | 7798 | 3'UTR | -102 | 91 | 21 |
|  | *E2F6* | 2213 | 3'UTR | -104 | 92 | 21 |
|  | *GRIA4* | 5501 | 3'UTR | -104 | 92 | 21 |
|  | *LPPR5* | 1328 | 3'UTR | -113 | 100 | 21 |
|  | *LYPD3* | 1608 | 3'UTR | -113 | 100 | 21 |
|  | *MDGA1* | 6044 | 3'UTR | -102 | 91 | 21 |
|  | *MPL* | 3526 | 3'UTR | -104 | 92 | 21 |
|  | *N4BP1* | 6992 | 3'UTR | -104 | 92 | 21 |
|  | *PLEKHF1* | 1447 | 3'UTR | -102 | 91 | 21 |
|  | *SLC16A7* | 11186 | 3'UTR | -104 | 92 | 21 |
|  | *URGCP* | 3699 | 5'UTR | -102 | 91 | 21 |
| miR-15b | *AGFG1* | 6199 | 3'UTR | -102 | 91 | 22 |
| bta-miR-17-3p | *ADAMTS1* | 1634 | CDS | -98 | 92 | 20 |
|  | *CNTNAP2* | 2530 | CDS | -98 | 92 | 20 |
|  | *LRRC28* | 616 | CDS | -98 | 92 | 20 |
|  | *LTBP3* | 1219 | CDS | -98 | 92 | 20 |
| bta-miR-181c | *KIAA1551* | 4861 | CDS | -110 | 90 | 24 |
| bta-miR-183 | *KLHL6* | 4491 | 3'UTR | -108 | 91 | 23 |
| bta-miR-185 | *ELL3* | 1006 | CDS | -110 | 95 | 22 |
|  | *PMVK* | 91 | 5'UTR | -106 | 91 | 22 |
| bta-miR-186 | *PNMA2* | 1292 | CDS | -102 | 91 | 22 |
|  | *RALA* | 324 | CDS | -102 | 91 | 22 |
|  | *ZCCHC12* | 635 | CDS | -102 | 91 | 22 |
| bta-miR-18a | *CNGA2* | 2981 | 3'UTR | -102 | 91 | 22 |
| bta-miR-190b | *PIAS2* | 1801 | CDS | -93 | 92 | 21 |
| bta-miR-196a | *C11orf68* | 885 | CDS | -102 | 91 | 22 |
|  | *HOXB8* | 1378 | 3'UTR | -110 | 98 | 22 |
|  | *SBNO1* | 905 | CDS | -106 | 94 | 22 |
|  | *SV2A* | 937 | CDS | -102 | 91 | 22 |
| bta-miR-196b | *HOXB8* | 1377 | 3'UTR | -110 | 93 | 23 |
| bta-miR-197 | *CELF5* | 2894 | 3'UTR | -110 | 91 | 22 |
|  | *CTAG2* | 497 | CDS | -113 | 93 | 22 |
|  | *DAAM2* | 3461 | 3'UTR | -110 | 91 | 22 |
|  | *DPF2* | 140 | CDS | -110 | 91 | 22 |
|  | *HEATR8* | 3035 | CDS | -113 | 93 | 22 |
|  | *MORC2* | 3697 | CDS | -113 | 93 | 22 |
|  | *MROH7* | 3035 | CDS | -113 | 93 | 22 |
|  | *SDK2* | 958 | CDS | -110 | 91 | 22 |
|  | *TMTC2* | 329 | 5'UTR | -113 | 93 | 22 |
| bta-miR-199a-3p | *SYNE1* | 12575 | CDS | -104 | 92 | 22 |
| bta-miR-199b | *KPNA6* | 641 | CDS | -108 | 91 | 23 |
| bta-miR-200b | *SSMEM1* | 555 | CDS | -98 | 92 | 21 |
| bta-miR-205 | *CDH26* | 2565 | CDS | -108 | 91 | 22 |
| bta-miR-222 | *PXMP4* | 1713 | 3'UTR | -104 | 92 | 21 |
|  | *KCNJ10* | 1505 | 3'UTR | -102 | 91 | 21 |
|  | *LRRC10* | 724 | CDS | -102 | 91 | 21 |
|  | *RNPEP* | 761 | CDS | -102 | 91 | 21 |
| bta-miR-26a | *CNBP* | 1014 | 3'UTR | -102 | 91 | 22 |
| bta-miR-26b | *NAP1L1* | 981 | CDS | -98 | 90 | 22 |
| bta-miR-27a-3p | *LYL1* | 1045 | CDS | -98 | 92 | 20 |
| bta-miR-27a-3p | *NLRC4* | 1423 | CDS | -98 | 92 | 20 |
| bta-miR-28 | *LPPR5* | 1324 | 3'UTR | -106 | 93 | 22 |
|  | *LYPD3* | 1605 | 3'UTR | -104 | 91 | 22 |
|  | *MDGA1* | 6040 | 3'UTR | -110 | 96 | 22 |
|  | *SLC16A7* | 11183 | 3'UTR | -106 | 93 | 22 |
|  | *TEX261* | 3248 | 3'UTR | -106 | 93 | 22 |
| bta-miR-29a | *LAMC2* | 2312 | CDS | -108 | 91 | 23 |
| bta-miR-30b-3p | *CHRNA2* | 692 | CDS | -104 | 94 | 21 |
| bta-miR-30b-3p | *IKZF3* | 1143 | CDS | -102 | 92 | 21 |
| bta-miR-31 | *ZFP41* | 3034 | 3'UTR | -104 | 92 | 21 |
|  | *FBLN2* | 2223 | CDS | -102 | 91 | 21 |
|  | *TPBG* | 1429 | CDS | -102 | 91 | 21 |
| bta-miR-320a | *ZFYVE1* | 4275 | 3'UTR | -108 | 91 | 22 |
| bta-miR-320b | *LILRB1* | 712 | CDS | -106 | 96 | 20 |
|  | *LILRB2* | 635 | CDS | -106 | 96 | 20 |
|  | *PHLPP1* | 5318 | CDS | -104 | 94 | 20 |
|  | *ITGB3* | 2995 | 3'UTR | -102 | 92 | 20 |
|  | *EXOC3L1* | 2800 | 3'UTR | -102 | 92 | 20 |
|  | *KDM6B* | 2890 | CDS | -102 | 92 | 20 |
|  | *LILRA5* | 507 | CDS | -102 | 92 | 20 |
|  | *LILRA6* | 493 | CDS | -102 | 92 | 20 |
|  | *LILRB1* | 713 | CDS | -102 | 92 | 20 |
|  | *LILRB3* | 480 | CDS | -102 | 92 | 20 |
| bta-miR-326 | *HEMK1* | 704 | CDS | -108 | 94 | 20 |
| bta-miR-326 | *TENC1* | 2862 | CDS | -108 | 94 | 20 |
| bta-miR-328 | *ENO4* | 419 | CDS | -121 | 97 | 22 |
|  | *RNF167* | 363 | 5'UTR | -115 | 92 | 22 |
|  | *WSCD2* | 2076 | CDS | -113 | 90 | 22 |
|  | *EMILIN1* | 216 | 5'UTR | -113 | 90 | 22 |
|  | *RHBDL1* | 20 | 5'UTR | -113 | 90 | 22 |
| bta-miR-330 | *TAS1R2* | 211 | CDS | -119 | 93 | 23 |
| bta-miR-331-3p | *CORO6* | 1507 | 3'UTR | -106 | 91 | 21 |
|  | *FGD2* | 1450 | CDS | -106 | 91 | 21 |
|  | *CORO6* | 1507 | 3'UTR | -106 | 91 | 21 |
|  | *FGD2* | 1450 | CDS | -106 | 91 | 21 |
| bta-miR-331-5p | *TSKU* | 1390 | 3'UTR | -93 | 96 | 18 |
| bta-miR-335 | *IL22* | 1051 | 3'UTR | -102 | 91 | 23 |
| bta-miR-345-3p | *ALMS1* | 10034 | CDS | -102 | 92 | 20 |
|  | *PRKCG* | 2249 | CDS | -104 | 94 | 20 |
|  | *PRRT3* | 1341 | CDS | -104 | 94 | 20 |
|  | *ZNF831* | 1564 | CDS | -102 | 92 | 20 |
| bta-miR-345-5p | *CRIP1* | 1 | 5'UTR | -104 | 91 | 21 |
|  | *CYTH4* | 2937 | 3'UTR | -104 | 91 | 21 |
|  | *DDI1* | 58 | 5'UTR | -104 | 91 | 21 |
|  | *DHODH* | 161 | CDS | -104 | 91 | 21 |
|  | *GABBR2* | 4462 | 3'UTR | -104 | 91 | 21 |
|  | *IBA57* | 1835 | 3'UTR | -104 | 91 | 21 |
|  | *LEPREL4* | 2529 | 3'UTR | -106 | 93 | 21 |
|  | *MDN1* | 7533 | CDS | -110 | 96 | 21 |
|  | *PHF13* | 1849 | 3'UTR | -104 | 91 | 21 |
|  | *SERPINB9* | 93 | 5'UTR | -104 | 91 | 21 |
|  | *TECPR2* | 7143 | 3'UTR | -106 | 93 | 21 |
|  | *YWHAB* | 260 | 5'UTR | -104 | 91 | 21 |
| bta-miR-346 | *CAMSAP2* | 97 | 5'UTR | -117 | 90 | 23 |
|  | *TENM4* | 35 | 5'UTR | -117 | 90 | 23 |
| bta-miR-34a | *GP5* | 1218 | CDS | -106 | 91 | 22 |
| bta-miR-34c | *LGR6* | 1293 | CDS | -106 | 93 | 22 |
| bta-miR-361 | *SYNRG* | 1671 | CDS | -104 | 91 | 22 |
| bta-miR-365-3p | *USP22* | 4323 | 3'UTR | -100 | 92 | 22 |
| bta-miR-370 | *CCDC83* | 84 | 5'UTR | -115 | 92 | 22 |
|  | *KRTAP13* | 63 | CDS | -113 | 90 | 22 |
|  | *TEP1* | 4901 | CDS | -113 | 90 | 22 |
| bta-miR-378 | *NFASC* | 9130 | 3'UTR | -110 | 93 | 22 |
|  | *TRABD2B* | 3323 | 3'UTR | -108 | 91 | 22 |
|  | *ZBTB20* | 113 | 5'UTR | -108 | 91 | 22 |
| bta-miR-379 | *DYRK2* | 1118 | CDS | -100 | 90 | 21 |
| bta-miR-381 | *SCAPER* | 3920 | CDS | -104 | 91 | 22 |
| bta-miR-383 | *FAT2* | 10586 | CDS | -106 | 93 | 22 |
|  | *GDF11* | 607 | CDS | -104 | 91 | 22 |
|  | *LRRIQ1* | 3376 | CDS | -104 | 91 | 22 |
| bta-miR-423-5p | *CREB3L1* | 1094 | CDS | -115 | 92 | 23 |
|  | *FMNL1* | 1866 | CDS | -115 | 92 | 23 |
|  | *FXYD3* | 1065 | 3'UTR | -113 | 90 | 23 |
|  | *RELL2* | 436 | 5'UTR | -113 | 90 | 23 |
|  | *SFSWAP* | 2705 | CDS | -113 | 90 | 23 |
|  | *SLFNL1* | 50 | 5'UTR | -115 | 92 | 23 |
|  | *SMOX* | 2280 | 3'UTR | -113 | 90 | 23 |
| bta-miR-424-3p | *GPR78* | 939 | CDS | -104 | 92 | 21 |
| bta-miR-424-3p | *ST18* | 103 | 5'UTR | -102 | 91 | 21 |
| bta-miR-432 | *CD177* | 1058 | CDS | -110 | 90 | 23 |
|  | *EPB41L1* | 339 | 5'UTR | -110 | 90 | 23 |
|  | *NTN1* | 4801 | 3'UTR | -110 | 90 | 23 |
|  | *PRSS57* | 373 | CDS | -113 | 91 | 23 |
|  | *RTL1* | 330 | CDS | -123 | 100 | 23 |
| bta-miR-450a | *MUC16* | 31487 | CDS | -100 | 92 | 22 |
| bta-miR-452 | *EPHB2* | 1408 | CDS | -104 | 91 | 22 |
|  | *HIVEP3* | 4350 | CDS | -104 | 91 | 22 |
|  | *NMD3* | 267 | CDS | -104 | 91 | 22 |
|  | *SEC61A1* | 1328 | CDS | -104 | 91 | 22 |
| bta-miR-484 | *CDC34* | 1164 | 3'UTR | -117 | 95 | 22 |
|  | *CTDP1* | 3042 | 3'UTR | -110 | 90 | 22 |
|  | *ESRRA* | 341 | CDS | -110 | 90 | 22 |
|  | *GHDC* | 1119 | CDS | -110 | 90 | 22 |
|  | *LOC100132703* | 137 | 5'UTR | -110 | 90 | 22 |
|  | *STK24* | 3234 | 3'UTR | -113 | 91 | 22 |
| bta-miR-487b | *NCKIPSD* | 1860 | CDS | -104 | 91 | 22 |
| bta-miR-491 | *MTOR* | 6826 | CDS | -113 | 93 | 22 |
| bta-miR-497 | *LRRC27* | 7042 | 3'UTR | -108 | 93 | 22 |
|  | *N4BP1* | 5036 | 3'UTR | -106 | 91 | 22 |
| bta-miR-503-5p | *ESRP2* | 1981 | CDS | -102 | 94 | 20 |
| bta-miR-505 | *RDH11* | 357 | CDS | -106 | 91 | 22 |
| bta-miR-628 | *ZNF552* | 913 | CDS | -100 | 90 | 22 |
| bta-miR-652 | *ANKS6* | 597 | CDS | -104 | 91 | 21 |
|  | *CLCN4* | 1318 | CDS | -104 | 91 | 21 |
|  | *DUPD1* | 501 | CDS | -104 | 91 | 21 |
|  | *FDX1* | 1588 | 3'UTR | -110 | 96 | 21 |
|  | *GPR179* | 1600 | CDS | -104 | 91 | 21 |
| bta-miR-671 | *GIPR* | 1236 | CDS | -121 | 92 | 23 |
|  | *SLC6A9* | 2144 | CDS | -121 | 92 | 23 |
|  | *TM9SF4* | 2738 | 3'UTR | -123 | 94 | 23 |
|  | *VAV2* | 3640 | 3'UTR | -121 | 92 | 23 |
|  | *VTI1A* | 2839 | 3'UTR | -121 | 92 | 23 |
|  | *VWA7* | 2626 | CDS | -121 | 92 | 23 |
|  | *ISLR* | 1564 | CDS | -119 | 90 | 23 |
|  | *PKN2* | 2066 | CDS | -119 | 90 | 23 |
|  | *SGIP1* | 1286 | CDS | -119 | 90 | 23 |
|  | *SLX4* | 3501 | CDS | -119 | 90 | 23 |
|  | *TBX5* | 1558 | CDS | -119 | 90 | 23 |
|  | *TRAF7* | 2630 | 3'UTR | -119 | 90 | 23 |
|  | *ZFHX3* | 10875 | CDS | -119 | 90 | 23 |
|  | *ZNF619* | 1962 | 3'UTR | -119 | 90 | 23 |
| bta-miR-7 | *ERP27* | 923 | 3'UTR | -108 | 91 | 24 |
| bta-miR-708 | *AQP2* | 4053 | 3'UTR | -113 | 93 | 23 |
|  | *DCAF10* | 7793 | 3'UTR | -110 | 91 | 23 |
|  | *HGSNAT* | 4092 | 3'UTR | -110 | 91 | 23 |
|  | *LIAS* | 142 | CDS | -110 | 91 | 23 |
|  | *MDGA1* | 6039 | 3'UTR | -110 | 91 | 23 |
| bta-miR-744 | *ANKZF1* | 1709 | CDS | -110 | 90 | 22 |
|  | *ARFRP1* | 1502 | 3'UTR | -110 | 90 | 22 |
|  | *CPNE9* | 104 | 5'UTR | -110 | 90 | 22 |
|  | *NEDD4L* | 7621 | 3'UTR | -110 | 90 | 22 |
| bta-miR-760-5p | *C1orf226* | 270 | CDS | -117 | 92 | 22 |
|  | *FSCN2* | 61 | 5'UTR | -117 | 92 | 22 |
|  | *SEPT5* | 238 | 5'UTR | -115 | 90 | 22 |
|  | *SLC35A2* | 1065 | 3'UTR | -115 | 90 | 22 |
|  | *SPTB* | 7601 | 3'UTR | -115 | 90 | 22 |
|  | *NOM1* | 1832 | CDS | -115 | 90 | 22 |
| bta-miR-874 | *PRKCH* | 273 | 5'UTR | -119 | 92 | 22 |
|  | *NADK* | 516 | CDS | -117 | 90 | 22 |
| bta-miR-877 | *ALOX12B* | 1513 | CDS | -104 | 92 | 20 |
|  | *TRERF1* | 1716 | CDS | -104 | 92 | 20 |
| bta-miR-92a | *EZR* | 1858 | CDS | -108 | 91 | 22 |
| bta-miR-92b | *MFRP* | 1204 | CDS | -110 | 90 | 22 |
|  | *MYO18A* | 5837 | CDS | -110 | 90 | 22 |
|  | *CIDEB* | 236 | 5'UTR | -110 | 90 | 22 |
| bta-miR-93 | *NOSTRIN* | 2566 | 3'UTR | -108 | 93 | 22 |
|  | *FZD5* | 1812 | CDS | -106 | 91 | 22 |
| bta-miR-935 | *ARNT* | 71 | 5'UTR | -117 | 90 | 23 |
|  | *FOXO3* | 558 | CDS | -119 | 92 | 23 |
|  | *LIMD2* | 10 | 5'UTR | -117 | 90 | 23 |
|  | *SAV1* | 255 | 5'UTR | -117 | 90 | 23 |
|  | *UFD1L* | 39 | 5'UTR | -117 | 90 | 23 |
| bta-miR-95 | *KCNS1* | 3587 | 3'UTR | -102 | 92 | 22 |
|  | *TMEM200B* | 817 | CDS | -100 | 90 | 22 |
|  | *TRIM47* | 2288 | 3'UTR | -100 | 90 | 22 |
|  | *ZNF497* | 3537 | 3'UTR | -100 | 90 | 22 |
| bta-miR-99a-3p | *CTAG1B* | 347 | CDS | -96 | 94 | 19 |
| bta-miR-99b | *DAPK3* | 1974 | 3'UTR | -110 | 90 | 22 |
|  | *UBP1* | 281 | 5'UTR | -110 | 90 | 22 |
