# Supplementary material for: Identification of Bovine miRNAs with the Potential to Affect Human Gene Expression
Source: Front Genet. 2022 Jan 11;12:705350. doi: 10.3389/fgene.2021.705350 (PMC8787201; doi:10.3389/fgene.2021.705350)
Supplement: Supplementary file 7 [file Table13.DOCX]

**Supplementary Table S9** Characteristics of interactions of bta-miRNA with human 5′UTR mRNA containing the BS clusters with length of 24 nt.

| **Gene** | **bta-miRNA** | **Start of**  **site, nt** | **ΔG,**  **kJ/mole** | **∆G/∆Gm_,_**  **%** | **Length,**  **nt** |
| --- | --- | --- | --- | --- | --- |
| *AFF2* | bta-miR-11975 | 14÷53 (14) | -117÷-121 | 92÷95 | 20 |
|  | bta-miR-11976 | 16÷52 (13) | -123÷-127 | 92÷95 | 21 |
|  | bta-mir-2885 | 22÷46 (9) | -110 | 93 | 19 |
|  | bta-miR-11976 | 101, 110 | -123 | 92 | 21 |
|  | bta-miR-11975 | 102, 111 | -117÷111 | 92-95 | 20 |
| *CUL3* | bta-miR-11976 | 197÷212 (6) | -121÷-127 | 90÷95 | 21 |
|  | bta-miR-11975 | 198÷213 (6) | -114÷-121 | 90÷95 | 20 |
|  | bta-miR-2885 | 200 | -110 | 93 | 19 |
| *FAM50A* | bta-miR-11975 | 78÷90 (5) | -115÷-121 | 90÷95 | 20 |
|  | bta-miR-11976 | 80÷89 (4) | -123÷-127 | 92÷95 | 21 |
|  | bta-miR-2885 | 80÷86 (3) | -110 | 93 | 19 |
| *GSK3B* | bta-miR-11976 | 9, 12 | -123÷-127 | 92÷95 | 21 |
|  | bta-miR-11975 | 10, 13 | -121 | 95 | 20 |
| *MAP2K3* | bta-miR-11976 | 117÷132 (4) | -118÷-127 | 92÷95 | 21 |
|  | bta-miR-11975 | 118÷133 (5) | -117÷-121 | 92÷95 | 20 |
|  | bta-miR-2885 | 126÷132 (3) | -110 | 93 | 19 |
| *MSI1* | bta-miR-11975 | 1÷16 (4) | -114÷-121 | 90÷95 | 20 |
|  | bta-miR-11976 | 9÷15 (3) | -123÷-127 | 92÷95 | 21 |
|  | bta-miR-2885 | 9÷15 (3) | -110 | 93 | 19 |
| *MTHFD1L* | bta-miR-11975 | 43÷49 (3) | -114÷-121 | 90÷95 | 20 |
|  | bta-miR-11976 | 45, 48 | -127 | 95 | 21 |
|  | bta-miR-2885 | 45, 48 | -110 | 93 | 19 |
| *NCKAP1* | bta-miR-11975 | 49÷64 (4) | -115÷-121 | 90÷95 | 20 |
|  | bta-miR-11976 | 60, 63 | -127 | 95 | 21 |
|  | bta-miR-2885 | 60, 63 | -110 | 93 | 19 |
| *RPRD2* | bta-miR-11976 | 138÷147 (3) | -127 | 95 | 21 |
|  | bta-miR-11975 | 139÷148 (4) | -117÷-121 | 90÷95 | 20 |
|  | bta-miR-2885 | 138÷147 (3) | -110 | 93 | 19 |
| *UBTF* | bta-miR-11975 | 31÷40 (3) | -117÷-121 | 92÷95 | 20 |
|  | bta-miR-11976 | 36, 39 | -121÷-127 | 90÷95 | 21 |
| *TCEA1* | bta-miR-11976 | 146÷152 (3) | -121÷-127 | 90÷95 | 21 |
|  | bta-miR-11975 | 147÷153 (3) | -115÷-121 | 90÷95 | 20 |
|  | bta-miR-2885 | 149, 152 | -110 | 93 | 19 |
| *THOC7* | bta-miR-11976 | 98÷104 (3) | -121÷-127 | 90÷95 | 20 |
|  | bta-miR-11975 | 99÷105 (3) | -115÷-121 | 95 | 20 |
|  | bta-miR-2885 | 98÷104 (3) | -110 | 93 | 19 |
| *USP7* | bta-miR-11976 | 44 | -123 | 92 | 21 |
|  | bta-miR-11975 | 45 | -117 | 92 | 20 |
|  | bta-miR-11975 | 94÷103 (4) | -121 | 95 | 20 |
|  | bta-miR-11976 | 96÷102 (3) | -121÷-127 | 90÷95 | 21 |
|  | bta-miR-2885 | 99, 102 | -110 | 93 | 19 |
| *ZNF219* | bta-miR-11976 | 275÷305 (4) | -127 | 95 | 21 |
|  | bta-miR-11975 | 276÷306 (5) | -115÷-121 | 90÷95 | 20 |
|  | bta-miR-2885 | 287, 290 | -110 | 93 | 19 |
