# Supplementary material for: Identification of Bovine miRNAs with the Potential to Affect Human Gene Expression
Source: Front Genet. 2022 Jan 11;12:705350. doi: 10.3389/fgene.2021.705350 (PMC8787201; doi:10.3389/fgene.2021.705350)
Supplement: Supplementary file 8 [file Table11.DOCX]

**Supplementary Table S7** Characteristics of interactions of bta-miRNA with human 5′UTR mRNA containing the BS clusters with length of 18 nt

| **Gene** | **bta-miRNA** | **Start of**  **site, nt** | **ΔG,**  **kJ/mole** | **∆G/∆Gm,**  **%** | **Length,**  **nt** |
| --- | --- | --- | --- | --- | --- |
| *ABCC1* | bta-miR-11976 | 27, 36 | -121÷-127 | 90÷95 | 21 |
|  | bta-miR-11975 | 28÷37 (3) | -117÷-121 | 92÷95 | 20 |
|  | bta-mir-2885 | 36 | -110 | 93 | 19 |
| *ASH1L* | bta-miR-11975 | 86, 89 | -117 | 92 | 20 |
| *BTF3L4* | bta-miR-11976 | 92, 98 | -123 | 92 | 21 |
|  | bta-miR-11975 | 93, 99 | -117 | 92 | 20 |
| *C2CD4C* | bta-miR-11975 | 36 | -117 | 92 | 20 |
| *CPT1A* | bta-miR-11976 | 95÷104 (4) | -121÷-123 | 90÷92 | 21 |
|  | bta-miR-11975 | 96÷105 (4) | -115÷-117 | 90÷92 | 20 |
| *EGLN1* | bta-miR-11975 | 3130÷3136 (3) | -117 | 92 | 20 |
|  | bta-miR-11976 | 3132, 3135 | -123 | 92 | 21 |
| *GRIN1* | bta-miR-11976 | 32 | -123 | 92 | 21 |
|  | bta-miR-11975 | 33 | -117 | 92 | 20 |
| *GTF2E2* | bta-miR-11976 | 54÷63 (4) | -123 | 92 | 21 |
|  | bta-miR-11975 | 55÷64 (3) | -117 | 92 | 20 |
| *MAST1* | bta-miR-11976 | 55÷ 67 (5) | -121÷-123 | 90÷92 | 21 |
|  | bta-miR-11975 | 56÷68 (5) | -114÷-116 | 90÷91 | 20 |
| *MEMO1* | bta-miR-11976 | 315 | -123 | 92 | 20 |
|  | bta-miR-11975 | 316 | -117 | 92 | 20 |
| *MPRIP* | bta-miR-11975 | 48÷60(5) | -114÷-117 | 90÷92 | 20 |
|  | bta-miR-11976 | 53÷59 (3) | -123 | 92 | 21 |
| *NOG* | bta-miR-11975 | 22, 25 | -115 | 90÷92 | 20 |
|  | bta-miR-11976 | 24 | -123 | 92 | 21 |
|  | bta-miR-2885 | 24 | -110 | 93 | 19 |
| *RIMS4* | bta-miR-11975 | 17÷23 (3) | -115 | 90 | 20 |
|  | bta-miR-11976 | 19, 22 | -121 | 90 | 21 |
| *RNF165* | bta-miR-11975 | 7÷19 (3) | -115 | 90 | 20 |
|  | bta-miR-11976 | 15 | -121 | 90 | 21 |
| *RNF220* | bta-miR-11975 | 108÷117 (4) | -117 | 92 | 20 |
|  | bta-miR-11976 | 110, 116 | -123 | 92 | 20 |
| *SCAP* | bta-miR-11976 | 103÷112 (3) | -121÷-127 | 92÷95 | 21 |
|  | bta-miR-11975 | 104÷113 (4) | -114÷-121 | 90÷95 | 20 |
|  | bta-miR-2885 | 103, 112 | -110 | 93 | 19 |
| *SEPHS1* | bta-miR-11976 | 161, 164 | -121 | 90 | 21 |
|  | bta-miR-11975 | 162÷165 (3) | -114÷-121 | 90÷95 | 20 |
|  | bta-miR-2885 | 167 | -110 | 93 | 19 |
| *SPEN* | bta-miR-11976 | 88÷97 (3) | -121÷-123 | 90÷92 | 21 |
|  | bta-miR-11975 | 89÷98 (3) | -115÷-117 | 90÷92 | 20 |
|  | bta-miR-2885 | 88 | -108 | 91 | 19 |
