# Supplementary material for: Identification of Bovine miRNAs with the Potential to Affect Human Gene Expression
Source: Front Genet. 2022 Jan 11;12:705350. doi: 10.3389/fgene.2021.705350 (PMC8787201; doi:10.3389/fgene.2021.705350)
Supplement: Supplementary file 9 [file Table6.DOCX]

**Supplementary Table S2** Characteristics of interactions of bta-miR-574-5p with human mRNA genes containing clusters of 1-13 binding sites

| **Gene** | **Start of first and**  **last sites, nt** | **ΔG,**  **kJ/mole** | **∆G/∆Gm_,_**  **%** |
| --- | --- | --- | --- |
| *AMOTL1* | 6022-6038 (9) | -115÷-119 | 90-93 |
| *ANK1* | 6551-6555 (3) | -117÷-121 | 92-95 |
| *ANKRD42* | 1887-1909 (12) | -115÷-119 | 90-93 |
| *ANO8* | 4032-4040 (4) | -119 | 93 |
| *APBA1* | 3554 | -115 | 90 |
| *ARHGAP35* | 7727-7739 (7) | -117÷-119 | 92-93 |
| *ARHGEF9* | 2538-2548 (6) | -119 | 93 |
| *ARID3B* | 3614,3616 | -115 | 90 |
| *ARRB1* | 6398-6418 (11) | -115÷-119 | 90-93 |
| *C15orf41* | 2383-2387 (3) | -115÷-119 | 90-93 |
| *CABP4* | 2762-2770 (5) | -115÷-119 | 90-93 |
| *CACNA2D2* | 4667 | -119 | 93 |
| *CACNG2* | 2899-2907 (4) | -115÷-117 | 90-92 |
| *CACNG2* | 3172 | -115 | 90 |
| *CALCOCO1* | 2553 | -117 | 92 |
| *CAMK2N1* | 1851-1863 (7) | -117÷-119 | 92-93 |
| *CARNS1* | 3224-3234 (6) | -117÷-119 | 92-93 |
| *CAMTA1* | 5316-5324 (4) | -115÷-117 | 90-92 |
| *CCND1* | 2594,2596 | -115÷-119 | 90,93 |
| *CD22* | 2730-2752 (10) | -115÷-121 | 90-95 |
| *CD93* | 3486-3498 (7) | -115÷-119 | 90-93 |
| *CDH12* | 3579 | -119 | 93 |
| *CDH6* | 4738- 4758 (11) | -115÷-119 | 90-93 |
| *CDK13* | 6265 | -115 | 90 |
| *CDKN2B* | 1745-1751(4) | -119 | 93 |
| *CDON* | 6343-6347 (3) | -115÷-119 | 90-93 |
| *CHRDL1* | 2248 – 2264 (9) | -115÷-119 | 90-93 |
| *CHST11* | 5216-5232 (9) | -115÷-119 | 90-93 |
| *CLIC6* | 2572-2586 (8) | -119 | 93 |
| *CNGA4* | 2097-2121(13) | -115÷-119 | 90-93 |
| *CNTNAP5* | 5192,5194 | -115 | 90 |
| *CPPED1* | 5407-5423 (9) | -115÷-119 | 90-93 |
| *CREB3L2* | 6067-6081 (8) | -117÷-119 | 92-93 |
| *CRHBP* | 1372-1384 (3) | -115 | 90 |
| *CYP4V2* | 2899-2923 (13) | -115÷-119 | 90-93 |
| *DCX* | 4207-4215 (4) | -115÷-119 | 90-93 |
| *DDHD1* | 6278-6288 (6) | -115÷-119 | 90-93 |
| *DGKG* | 2920, 2930 | -115 | 90 |
| *DMD* | 11763-11771(5) | -115 | 90 |
| *DNAJC15* | 1127-1140 (7) | -115÷-119 | 90-93 |
| *DNAJC6* | 5545-5566 (9) | -115÷-119 | 90-93 |
| *DOCK1* | 6542 | -115 | 90 |
| *DOK6* | 7828-7852 (13) | -115÷-119 | 90-93 |
| *DPYSL5* | 2929-2950 (11) | -115÷-119 | 90-93 |
| *E2F8* | 3285-3293 (5) | -115÷-119 | 90-93 |
| *EDA2R* | 1439,1441 | -115 | 90 |
| *EFNB1* | 2525-2529 (3) | -119 | 93 |
| *EHD3* | 2273-2283(6) | -115÷-119 | 90-93 |
| *EN2* | 2561 | -121 | 95 |
| *ENPP5* | 1775-1781(4) | -115 | 90 |
| *EOGT* | 2681-2693(7) | -115÷-119 | 90-93 |
| *ERP27* | 988-996 (5) | -117÷-119 | 92-93 |
| *ESCO2* | 1990-2012 (7) | -115÷-117 | 90-92 |
| *FAM117B* | 2079-2103(13) | -117÷-119 | 92-93 |
| *FAM123B* | 4044-4056 (7) | -115÷-119 | 90-93 |
| *FAM163A* | 2109-2123 (8) | -115÷-119 | 90-93 |
| *FAM167A* | 3151-3179 (10) | -115÷-119 | 90-93 |
| *FAM83C* | 2510-2522(7) | -115÷-119 | 90-93 |
| *FAM84A* | 5412, 5414 | -115÷-119 | 90,93 |
| *FBRSL1* | 4179-4189 (6) | -115÷-119 | 90-93 |
| *FLNC* | 8429-8440 (6) | -115÷-119 | 90-93 |
| *FLRT2* | 6830 | -115 | 90 |
| *FLVCR1* | 3923 | -115 | 90 |
| *FOXI2* | 1880-2112 (9) | -115÷-117 | 90-92 |
| *FREM2* | 15371 | -115 | 90 |
| *FUT9* | 4398-4418 (7) | -115÷-117 | 90-92 |
| *GABRB2* | 6017-6027 (3) | -115÷-123 | 90-97 |
| *GABRB3* | 4107-4111(3) | -115÷-119 | 90-93 |
| *GIMAP1* | 2024-2032 (5) | -119 | 93 |
| *GLP1R* | 1547-1567 (11) | -115÷-119 | 90-93 |
| *GLYR1* | 1964-1978 (8) | -119 | 93 |
| *GNE* | 3400-3406 (4) | -115÷-119 | 90-93 |
| *GPATCH2L* | 2220-2224 (3) | -115÷-119 | 90-93 |
| *GPBP1L1* | 3012,3022 | -117 | 92 |
| *GPR173* | 3406,3408 | -117 | 92 |
| *GPR83* | 2063 | -119 | 93 |
| *GRIA3* | 3350-3366 (9) | -115÷-119 | 90-93 |
| *GRIA4* | 5083-5097(8) | -119 | 93 |
| *GRID1* | 3215 | -117 | 92 |
| *GYPA* | 1134,1136 | -115 | 90 |
| *HPS3* | 3252-3268 (9) | -115÷-119 | 90-93 |
| *HS3ST4* | 2384,2388 | -117 | 92 |
| *HS6ST3* | 3848-3854 (4) | -119 | 93 |
| *IFFO2* | 4394-4408 (8) | -115÷-121 | 90-95 |
| *IGF1* | 4043-4061(10) | -115÷-119 | 90-93 |
| *IGF2* | 2289-2730 (8) | -115÷-119 | 90-93 |
| *IGLON5* | 2081-2083 (3) | -115÷-121 | 90-95 |
| *INHBA* | 2038-2052 (8) | -115÷-119 | 90-93 |
| *IPO9* | 4610-4616 (3) | -117÷-119 | 92-93 |
| *ITGAM* | 4455,4457 | -117 | 92 |
| *JAKMIP2* | 7208,7210 | -119 | 93 |
| *JARID2* | 4737 | -117 | 92 |
| *KAT6A* | 7920-7928 (5) | -115÷-119 | 90-93 |
| *KATNAL1* | 4396-4410 (8) | -115÷-119 | 90-93 |
| *KATNAL1* | 4525-4550 (4) | -115 | 90 |
| *KCNIP3* | 1006-1024 (10) | -115÷-119 | 90-93 |
| *KCNJ15* | 2912 | -115 | 90 |
| *KCNK10* | 6167-6195 (8) | -115÷-119 | 90-93 |
| *KCNQ3* | 3561-3581(11) | -115÷-119 | 90-93 |
| *KIAA0141* | 4869-4875 (4) | -115÷-117 | 90-92 |
| *KIAA1211* | 4403 | -117 | 92 |
| *KIAA1549L* | 9103-9119 (9) | -115÷-119 | 90-93 |
| *KIAA2018* | 9952-9966 (8) | -115÷-119 | 90-93 |
| *KLF7* | 1359-1373 (3) | -115 | 90 |
| *LCN12* | 1012 | -117 | 92 |
| *LEPREL1* | 2948-2962 (8) | -119 | 93 |
| *LHFPL5* | 1460-1478 (10) | -115÷-119 | 90-93 |
| *LHFPL5* | 1520-11532 (7) | -115÷-119 | 90-93 |
| *LMOD3* | 2730 | -117 | 92 |
| *LMX1B* | 5353 | -121 | 95 |
| *LPP* | 14328-14352 (13) | -115÷-119 | 90-93 |
| *LRP2BP* | 3994-4012 (9) | -115 | 90 |
| *LRTM2* | 2843,2845 | -115÷-123 | 90,97 |
| *LRTM2* | 2948-2972 (13) | -115÷-119 | 90-93 |
| *LYRM7* | 5254-5278 (13) | -115÷-119 | 90-93 |
| *MAF* | 2103-2126 (12) | -115÷-121 | 90-95 |
| *MAP2* | 8793-8808 (8) | -115÷-119 | 90-93 |
| *MAP3K2* | 2665-2669 (3) | -117÷-119 | 92-93 |
| *MARCH4* | 3768-3784 (9) | -117÷-119 | 92-93 |
| *MCM8* | 2995-3019 (12) | -117÷-119 | 92-93 |
| *MEF2C* | 4441 | -115 | 90 |
| *MEGF8* | 9263 | -115 | 90 |
| *MGRN1* | 3075-3083 (5) | -115÷-119 | 90-93 |
| *MNT* | 4276-4302 (11) | -115÷-119 | 90-93 |
| *MXD1* | 4535-4539 (3) | -115 | 90 |
| *MYLK4* | 3449-3453 (3) | -119÷-121 | 93-95 |
| *MYO5A* | 9636-9644 (5) | -115 | 90 |
| *NAV1* | 7556-7578 (12) | -119 | 93 |
| *NCAM1* | 3099,3125 | -115÷-119 | 90-93 |
| *NCK2* | 2243 | -119 | 93 |
| *NEUROD2* | 2888 | -119 | 93 |
| *NLK* | 2144-2152 (5) | -119 | 93 |
| *NOS1* | 12000-12015 (8) | -115÷-119 | 90-93 |
| *NR4A2* | 2605-2615 (6) | -115 | 90 |
| *PIK3R5* | 3374 | -119 | 93 |
| *PLEKHA6* | 7024 | -115 | 90 |
| *POU4F2* | 2526-2532 (4) | -115÷-119 | 90-93 |
| *PPARA* | 9025-9035 (6) | -115÷-119 | 90-93 |
| *PPP2R1B* | 4562-4580 (10) | -119 | 93 |
| *PRKCI* | 3460 | -115 | 90 |
| *PTCHD1* | 2901-921 (11) | -117÷-119 | 92-93 |
| *RAB3IP* | 4085-4099 (8) | -115÷-119 | 90-93 |
| *RAB7A* | 1009-1030 (8) | -115÷-119 | 90-93 |
| *RALGAPB* | 7475-7489 (8) | -115-121 | 90-95 |
| *RBBP9* | 1399 | -115 | 90 |
| *RCVRN* | 881,883 | -115 | 90 |
| *REEP5* | 1477-1495 (10) | -115÷-119 | 90-93 |
| *RGS4* | 1780-1788 (5) | -115÷-119 | 90-93 |
| *RIMS1* | 5688 | -117 | 92 |
| *RPH3A* | 2858-2868 (6) | -115÷-119 | 90-93 |
| *RSPO1* | 1902-1919 (8) | -115÷-117 | 90-92 |
| *SAMD12* | 7786-7796 (6) | -115÷-119 | 90-93 |
| *SAMD9L* | 6480-6507 (13) | -115÷-119 | 90-93 |
| *SDK2* | 7576-7585 (5) | -115÷-119 | 90-93 |
| *SDK2* | 8023-8035 (7) | -115÷-119 | 90-93 |
| *SEMA5B* | 4687-4697 (3) | -115÷-121 | 90-95 |
| *SEMA6A* | 4126-4153 (4) | -115 | 90 |
| *SENP1* | 2962 | -115 | 90 |
| *SH3TC2* | 20875-20897 (12) | -119 | 93 |
| *SHB* | 3404-3480 (11) | -115÷-119 | 90-93 |
| *SIGMAR1* | 1536-1557 (10) | --115÷-119 | 90-93 |
| *SIN3A* | 4924 | -115 | 90 |
| *SLC23A2* | 4903, 4905 | -119÷-121 | 93-95 |
| *SLC2A12* | 5530,5532 | -115÷-119 | 90-93 |
| *SLC31A1* | 866-878 (7) | -115÷-119 | 90-93 |
| *SLC43A3* | 1987,1989 | -119 | 93 |
| *SLC9A8* | 3113 | -117 | 92 |
| *SLCO4C1* | 2718-2732 (4) | -115÷-119 | 90-93 |
| *SLITRK3* | 4414-4436 (12) | -115÷-123 | 90-97 |
| *SMAD4* | 7739-7829 (9) | -115÷-119 | 90-93 |
| *SNAI2* | 1110,1112 | -115 | 90 |
| *SNX2* | 1736-1752 (9) | -115÷-119 | 90-93 |
| *SPN* | 2931-2943 (7) | -115÷-119 | 90-93 |
| *SPRY4* | 3457-3463 (4) | -115÷-119 | 90-93 |
| *SPTLC3* | 2182 | -119 | 93 |
| *SRD5A3* | 1414-1432 (10) | -119 | 93 |
| *SRGAP2* | 3985-4002 (6) | -115÷-119 | 90-93 |
| *SSX1* | 1067-1081(8) | -115÷-119 | 90-93 |
| *SSX4B* | 910-919 (4) | -115÷-119 | 90-93 |
| *SSX4* | 1035-1044 (4) | -115÷-119 | 90-93 |
| *SSX5* | 1196-1218 (12} | -115÷-121 | 90-95 |
| *ST18* | 5196 | -117 | 92 |
| *STXBP5L* | 6699 | -119 | 93 |
| *STXBP6* | 2560-2572 (7) | -119 | 93 |
| *TCTE1* | 2239-2267 (15) | -115÷-119 | 90-93 |
| *THSD7A* | 7941 | -117 | 92 |
| *TIPRL* | 2535 | -119 | 93 |
| *TMEM130* | 2002-2025 (12) | -117÷-121 | 92-95 |
| *TMEM150C* | 921,923 | -115÷-119 | 90-93 |
| *TMEM35* | 1034 | -115 | 90 |
| *TRAF3IP2* | 5390 | -115 | 90 |
| *TRIOBP* | 7487-7504 (9) | -115÷-119 | 90-93 |
| *TSTD3* | 1900,1902 | -115÷-119 | 90-93 |
| *TUSC5* | 3058-3064 (4) | -115÷-117 | 90-92 |
| *TXNL4B* | 1189,1191 | -117 | 92 |
| *UBN2* | 8985-8989 (3) | -117÷-123 | 92-97 |
| *VAMP4* | 1891-1899 (5) | -117÷-119 | 92-93 |
| *VGLL3* | 5334 | -115 | 90 |
| *VSNL1* | 1022-1044 (12) | -119 | 93 |
| *WNT4* | 1982-2000 (10) | -117÷-119 | 92-93 |
| *XG* | 2188 | -117 | 92 |
| *XRCC1* | 2034-2054 (10) | -115÷-119 | 90-93 |
| *ZC3H12D* | 2582-2588 (4) | -119 | 93 |
| *ZDHHC15* | 5123-5137 | -115÷-119 | 90-93 |
| *ZDHHC3* | 11015 | -121 | 95 |
| *ZEB1* | 3588-3604 (9) | -119 | 93 |
| *ZFP92* | 3288-3308 (11) | -115÷-119 | 90-93 |
| *ZNF382* | 1913-1917 (3) | -119 | 93 |
| *ZNF529* | 2579-2586 (3) | -119 | 93 |
| *ZNF618* | 2953-2975 (12) | -115÷-119 | 90-93 |
| *ZNF655* | 3958,3962 | -115÷-117 | 90-92 |
| *ZRANB1* | 3794-3806 (7) | -115÷-119 | 90-93 |
| *ZSWIM1* | 1632-1642 (9) | -115÷-119 | 90-93 |
