# Supplementary material for: Identification of Bovine miRNAs with the Potential to Affect Human Gene Expression
Source: Front Genet. 2022 Jan 11;12:705350. doi: 10.3389/fgene.2021.705350 (PMC8787201; doi:10.3389/fgene.2021.705350)
Supplement: Supplementary file 10 [file Table14.DOCX]

**Supplementary Table S10** Characteristics of interactions of bta-miRNA with human 5′UTR mRNA containing the BS clusters with length of 27 – 45 nt.

| Gene | bta-miRNA | Start of  site, nt | ΔG,  kJ/mole | ∆G/∆Gm_,_  % | Length,  nt |
| --- | --- | --- | --- | --- | --- |
| *SBF1* | bta-miR-11976 | 41÷50 (4) | -121÷-127 | 90÷95 | 21 |
|  | bta-miR-11975 | 42÷51 (4) | -115÷-121 | 90÷95 | 20 |
|  | bta-miR-2885 | 44÷50 (3) | -110 | 93 | 19 |
| *SMAD9* | bta-miR-11976 | 56÷65 (4) | -121÷-127 | 90÷95 | 21 |
|  | bta-miR-11975 | 57÷66 (4) | -117÷-121 | 92÷95 | 20 |
|  | bta-miR-2885 | 59÷65 (3) | -110 | 93 | 19 |
| *BCL11A* | bta-miR-11975 | 183÷192 (4) | -115÷-121 | 90÷95 | 20 |
|  | bta-miR-11976 | 185÷191 (3) | -127 | 95 | 21 |
| *WBP4* | bta-miR-11975 | 76÷91 (6) | -117÷-121 | 92÷95 | 20 |
|  | bta-miR-11976 | 78÷90 (5) | -123÷-127 | 92÷95 | 21 |
|  | bta-miR-2885 | 81÷90 (4) | -110 | 93 | 19 |
| *GNB2* | bta-miR-11976 | 75÷98 (9) | -121÷-127 | 90÷95 | 21 |
|  | bta-miR-11975 | 76÷99 (9) | -114÷-121 | 90÷95 | 20 |
| *KIF3B* | bta-miR-11976 | 49÷73 (9) | -114÷-127 | 90÷95 | 21 |
|  | bta-miR-11975 | 50÷74 (9) | -114÷-121 | 90÷95 | 20 |
|  | bta-miR-2885 | 52÷73 (7) | -110 | 93 | 19 |
| *NDRG3* | bta-miR-11976 | 9÷39 (9) | -121÷-127 | 90÷95 | 21 |
|  | bta-miR-11975 | 10÷40 (9) | -115÷-121 | 90÷95 | 20 |
|  | bta-miR-2885 | 18÷39 (8) | -110 | 93 | 19 |
| *BCL2L11* | bta-miR-11975 | 61÷88 (10) | -117÷-121 | 92÷95 | 20 |
|  | bta-miR-11976 | 63÷87 (9) | -123÷-127 | 92÷95 | 21 |
| *RHOT1* | bta-miR-11975 | 1÷28 (10) | -121 | 95 | 20 |
|  | bta-miR-11976 | 3÷27 (9) | -127 | 95 | 21 |
|  | bta-miR-2885 | 3÷27 (9) | -110 | 93 | 19 |
