# Supplementary material for: Identification of Bovine miRNAs with the Potential to Affect Human Gene Expression
Source: Front Genet. 2022 Jan 11;12:705350. doi: 10.3389/fgene.2021.705350 (PMC8787201; doi:10.3389/fgene.2021.705350)
Supplement: Supplementary file 11 [file Table10.DOCX]

**Supplementary Table S6** Characteristics of interactions of bta-miRNA with human CDS mRNA containing the BS clusters with length of 27-36 nt

| **Gene** | **bta-miRNA** | **Start of**  **site, nt** | **ΔG, kJ/mole** | **∆G/∆Gm_,_**  **%** | **Length,**  **nt** |
| --- | --- | --- | --- | --- | --- |
| *DMRTA2* | bta-miR-11975 | 1502÷1511 (4) | -114÷-121 | 90÷95 | 20 |
|  | bta-miR-11976 | 1504÷1510 (4) | -121÷-127 | 90÷95 | 21 |
|  | bta-miR-2885 | 1504÷1513 (4) | -110 | 92 | 19 |
| *DLX6* | bta-miR-11976 | 131÷143 (5) | -121-127 | 90-95 | 21 |
|  | bta-miR-11975 | 132÷144 (5) | -115-121 | 90-95 | 20 |
| *FOXF2* | bta-miR-11976 | 210÷216 (3) | -127 | 95 | 21 |
|  | bta-miR-11975 | 211÷217 (3) | -121 | 95 | 20 |
|  | bta-miR-2885 | 210÷216 (3) | -110 | 93 | 19 |
| *IRF2BPL* | bta-miR-11976 | 1363÷-1375(5) | -123÷-127 | 92÷95 | 21 |
|  | bta-miR-11975 | 1364÷1376 (5) | -117 | 92 | 20 |
|  | bta-miR-2885 | 1369÷1375 (3) | -110 | 93 | 19 |
| *MNX1* | bta-miR-11975 | 670÷685 (6) | -117÷-121 | 92 | 20 |
|  | bta-miR-11976 | 672÷684 (5) | -123÷-127 | 92÷95 | 21 |
|  | bta-miR-2885 | 678÷684 (3) | -110 | 93 | 19 |
| *NKX2-3* | bta-miR-11976 | 1009÷1024 (6) | -121÷-127 | 90÷95 | 21 |
|  | bta-miR-11975 | 1010÷1025 (6) | -115÷-121 | 95 | 20 |
|  | bta-miR-2885 | 1018÷1024 (3) | -110 | 93 | 19 |
| *ZNF703* | bta-miR-11975 | 1711÷1722 (5) | -115÷-121 | 90÷95 | 20 |
|  | bta-miR-11976 | 1712÷1721 (4) | -121÷-127 | 90÷95 | 21 |
|  | bta-miR-2885 | 1712÷1718 (3) | -110 | 93 | 19 |
| *CASKIN1* | bta-miR-11975 | 4056÷4077 (6) | -115÷-127 | 90÷95 | 20 |
|  | bta-miR-11976 | 4064÷4076 (5) | -121÷-127 | 90÷95 | 21 |
|  | bta-miR-2885 | 4067÷4076 (4) | -110 | 93 | 19 |
| *ZSWIM6* | bta-miR-11975 | 505÷532 (10) | -115÷-121 | 90÷95 | 20 |
|  | bta-miR-11976 | 507÷531 (3) | -121÷-127 | 90÷95 | 21 |
|  | bta-miR-2885 | 507 | -112 | 94 | 19 |
| *FOXE1* | bta-miR-11975 | 1159÷1177 (6) | -117÷-121 | 92÷95 | 20 |
|  | bta-miR-11976 | 1167÷1176 (4) | -127 | 95 | 21 |
|  | bta-miR-2885 | 1167÷1176 (4) | -110 | 93 | 19 |
| *ZIC5* | bta-miR-2885 | 1316 | -117 | 98 | 19 |
|  | bta-miR-11976 | 1316 | -134 | 100 | 21 |
|  | bta-miR-11975 | 1317, 1326 | -114÷-127 | 90÷100 | 20 |
|  | bta-miR-11975 | 1467÷1497(10) | -115÷-121 | 90÷95 | 20 |
|  | bta-miR-11976 | 1475÷1496 (8) | -123÷-127 | 92÷95 | 21 |
|  | bta-miR-2885 | 1478÷1490 (5) | -110 | 93 | 19 |
